# Clinical, Virologic, and Immunologic Characteristics of Zika Virus Infection in a Cohort of US Patients: Prolonged RNA Detection in Whole Blood

**DOI:** 10.1093/ofid/ofy352

**Published:** 2018-12-19

**Authors:** Hana M El Sahly, Rodion Gorchakov, Lilin Lai, Muktha S Natrajan, Shital M Patel, Robert L Atmar, Wendy A Keitel, Daniel F Hoft, Jill Barrett, Jason Bailey, Srilatha Edupuganti, Vanessa Raabe, Henry M Wu, Jessica Fairley, Nadine Rouphael, Kristy O Murray, Mark J Mulligan

**Affiliations:** 1 Department of Molecular Virology and Microbiology, Baylor College of Medicine, Houston, Texas; 2 Section of Pediatric Tropical Medicine, Center for Human Immunobiology, Department of Pediatrics, Baylor College of Medicine, Houston, Texas; 3 Hope Clinic of the Emory Vaccine Center, Division of Infectious Diseases, Department of Medicine, School of Medicine, Emory University, Decatur, Georgia; 4 Section of Infectious Diseases, Department of Medicine, Baylor College of Medicine, Houston, Texas; 5 Departments of Molecular Microbiology & Immunology and Internal Medicine, Division of Infectious Diseases, Allergy & Immunology, Saint Louis University, St. Louis, Missouri; 6 The Emmes Corporation, Rockville, Maryland; 7 Division of Infectious Diseases, Department of Medicine, School of Medicine, Emory University, Decatur, Georgia; 8 Division of Infectious Diseases and Microbiology and NYU Langone Vaccine Center, New York University, New York City, New York

**Keywords:** Zika, diagnostics, serologic response, cell-mediated immune response, dengue

## Abstract

**Background:**

Clinical, virologic, and immunologic characteristics of Zika virus (ZIKV) infections in US patients are poorly defined.

**Methods:**

US subjects with suspected ZIKV infection were enrolled. Clinical data and specimens were prospectively collected for ZIKV RNA detection and serologic and cellular assays. Confirmed ZIKV infection (cases) and ZIKV-negative (controls) subjects were compared. Dengue-experienced and dengue-naïve cases were also compared.

**Results:**

We enrolled 45 cases and 14 controls. Commonly reported symptoms among cases and controls were maculopapular rash (97.8% and 81.8%), fatigue (86.7% and 81.8%), and arthralgia (82.2% and 54.5%), respectively. The sensitivity (94%) and duration of infection detection (80% positivity at 65–79 days after disease onset) by polymerase chain reaction were highest in whole-blood specimens. ZIKV-neutralizing antibodies had a half-life of 105 days and were significantly higher in dengue virus–experienced cases than naïve ones (*P* = .046). In intracellular cytokine staining assays, the ZIKV proteins targeted most often by peripheral blood mononuclear cells from cases were structural proteins C and E for CD4+ T cells and nonstructural proteins NS3, NS5, and NS4B for CD8+ T cells.

**Conclusions:**

ZIKV RNA detection was more frequent and prolonged in whole-blood specimens. Immunoglobulin G (IgG) and neutralizing antibodies, but not IgM, were influenced by prior dengue infection. Robust cellular responses to E and nonstructural proteins have potential vaccine development implications.

Zika virus (ZIKV), a member of the *Flaviviridae* family, has been known to infect humans for 7 decades, with sporadic reports of disease and, until recently, no known complications. Most ZIKV infections have been subclinical, with much of the literature on human infections resulting from serosurveillance studies [1–3]. However, since 2007, the description of the disease pattern caused by ZIKV has changed dramatically in at least 2 ways. First, intense epidemics with high attack rates have occurred: on Yap Island in 2007 and in French Polynesia in 2013, followed shortly thereafter by large epidemics in South, Central, and North American countries and in the Caribbean [4–6]. Second, serious clinical sequelae of ZIKV infections have been documented, including congenital neurologic abnormalities when infections occur during pregnancy and neurologic complications, especially Guillain Barré syndrome (GBS), when infections occur in adults or children [7, 8]. The spectrum of neurologic manifestations associated with congenital Zika syndrome is wide [9]. In a study that included 2549 pregnancies during which the pregnant women had evidence of possible ZIKV infection, 122 (5%) fetuses had birth defects, mostly microcephaly (89%) and other brain abnormalities [10]. The spectrum of adult neurologic complications includes GBS, encephalitis, transverse myelitis, and chronic inflammatory demyelinating polyneuropathy [11]. These complications are in part due to the neurotropism of the virus [12–14]. In addition to mosquito-borne and vertical transmission of the virus, recent outbreaks have established sexual intercourse as a mode of transmission, with implications for conception planning and behavioral counseling [15]. *Aedes aegypti*, the mosquito vector of the ZIKV, dengue, chikungunya, and yellow fever viruses, is present in the United States, mostly in the southern half of the country. However, thus far, the local epidemiologic and socioeconomic conditions have not been favorable for sustained transmission, leading to large outbreaks of these viruses in the contiguous United States.

Evaluation of the clinical, virologic, and immunologic characteristics of ZIKV infection has occurred in regions hard hit by the epidemic. We set out to describe the clinical, virologic, and immune response features of ZIKV infection in patients living in the United States who acquired the infection while traveling or during the limited vector-borne transmission events in Miami, Florida. The planned interim analysis data are presented in this report.

## METHODS

### Study Design

This was a prospective, observational cohort study of persons living in the contiguous United States who were 15–70 years of age and had suspected ZIKV infection. The purpose of the study was to evaluate the clinical spectrum of the disease, the presence and persistence of ZIKV RNA in body fluids, and the humoral and cellular immune responses to ZIKV infection, as well as create a repository of samples for future research use. Subjects were recruited at Baylor College of Medicine in Houston, Texas, and Emory University in Atlanta, Georgia, using passive referrals and active case-finding from health care facilities and laboratory-based screening. The study was approved by the respective institutional review boards. All subjects provided written informed consent and were followed for up to 12 months, depending on the time elapsed between disease onset and enrollment. At baseline, medical, sexual, and travel histories, physical exam, and blood, urine, and saliva specimens were obtained from subjects. In addition, subjects had the option to give semen, vaginal secretions, and/or breast milk specimens.

### Definitions

A person was suspected of having Zika infection based on clinical presentation and history of ZIKV exposure (travel to endemic area or sexual exposure). ZIKV infection suspects who had confirmed infection based the detection of ZIKV RNA in a body fluid specimen or had positive ZIKV immunoglobulin M (IgM) and NAb in serum with lower/no NAb against dengue virus (DENV) 1–4 (given the high cross-reactivity between the 2 viruses) were defined as the study “cases.” Suspects who did not meet the definition of a case were followed up as test-negative “controls.” The timing of medical history and specimen collection were indicated by days post onset of symptoms (DPO), with the first reported day of symptoms designated as DPO 1. The number of visits for each subject depended on the DPO upon enrollment, with a maximum of 7 visits through 1 year. Symptoms’ severity grading was as follows: Symptoms were graded as mild if they did not interfere with daily activity, moderate if they resulted in some interference with daily activity, and severe if they interfered significantly with daily activity. Based on an analysis of the serological and epidemiologic data available, DENV-experienced subjects were defined as those with evidence of baseline DENV-specific immunity (DENV-specific NAb titers ≥250). DENV-naïve subjects were defined as those with no or only low-level cross-reactive DENV Ab titers (<250) [16]. The study clinical population was defined as all symptomatic subjects. The study immunogenicity population was defined as all subjects with evaluable blood samples.

### ZIKV RNA Detection

Serum and urine supernatant aliquots of 200 µL were stored at –70°C, whereas whole-blood packed cells, saliva, vaginal secretions, semen, and breast milk were mixed with lysis buffer with carrier RNA (Qiagen, Valencia, CA) before storing at –70°C. RNA was extracted using the QIAamp MinElute Virus Spin kit (Qiagen) following the original manufacturer protocol (serum) or with optimizations. Modifications included pretreatment of urine supernatant with Urine Conditioning Buffer (Zymo Research, Irvine, CA), and adjusted incubation with elution buffer (5 minutes at 56°C) for the remaining specimen types. In addition to the latter, RLT Plus buffer and RNeasy MinElute Spin Columns (Qiagen) were used during semen sample extractions. ZIKV RNA in the samples was detected by reverse transcription polymerase chain reaction (RT-PCR) using TaqMan Fast Virus 1-Step Master Mix on a ViiA 7 RT- PCR System (ThermoFisher Scientific, Waltham, MA) with a previously described primers/probe set [17]. Reactions were performed in duplicate with appropriate positive and negative controls, and a standard curve was developed based on a synthetic RNA fragment of the ZIKV genome to estimate the number of genome copy equivalents in the samples. Specimens with average Ct values above the lowest point of the standard curve but below Ct 40 were interpreted as “positive, below limit of quantification.” Samples with repeated discordant duplicates (positive and negative) were interpreted as “indeterminate.” Separate reactions were run in parallel with a primers/probe set detecting the human RNase P gene to control for proper nucleic acids extraction and absence of PCR inhibition [18].

### Viral Stocks for Focus Reduction Neutralization Test and IgM Antibody Capture Enzyme-Linked Immunosorbent Assay

The ZIKV strain used in these assays (PRVABC59 [KU501215.1]) was provided by the US Centers for Disease Control and Prevention. DENV1 (Hawaii), DENV2 (New Guinea C), and DENV4 (H241) viruses were provided by BEI Resources (https://www.beiresources.org). DENV3 (Sleman/78) was provided by Jens Wrammert, PhD (Emory University). Additional details of viral stock production are in the Supplementary Data. Titers of the passaged viruses were determined by focus forming assay [19]. For the Zika IgM antibody capture enzyme-linked immunosorbent assay (MAC-ELISA), ZIKV stocks were inactivated with 0.1% beta-propiolactone at 4°C overnight. Antigen was produced by centrifuging inactivated ZIKV and mock Vero cell supernatants in Amicon Ultra-15 Centrifugal Filters at 3500×*g* for 25 minutes at 4°C. Prepared antigen was stored at –80°C until use. Serum anti-ZIKV IgM Abs were detected by the Zika MAC-ELISA, as previously described [20, 21]. To detect Zika IgG binding antibodies, the MAC-ELISA was adapted from previous work and modified by coating with antihuman IgG (01-10-06, KPL) at 1:500 dilution [17]. Serum NAbs against ZIKV or DENV1–4 were measured by focus reduction neutralization test (FRNT), as previously described [19] with modifications (Supplementary Data). Foci were imaged and counted using a CTL-Immunospot S6 Micro Analyzer. FRNT_50_ titers were determined using GraphPad Prism software.

### Intracellular Cytokine Staining Assays

A total of 706 15-mer peptides that overlapped by 11-mers spanning the entire proteome of ZIKV (PRVABC59, Gene Bank accession #KU501215) and 306 overlapping peptides spanning the DENV2 E, NS3, and NS5 proteins were synthesized. These peptides were reconstituted in DMSO and then combined into pools that represented each of the 10 ZIKV and 3 DENV2 proteins. Details of the ZIKV and DENV2 peptide pools are available in the Supplementary Data. To detect interferon (IFN)-γ, interleukin (IL)-2, tumor necrosis factor (TNF)–α, CD107a, and MIP-1-ß production in response to ZIKV peptides, subjects’ cryopreserved peripheral blood mononuclear cells (PBMCs) were thawed and rested overnight, and then incubated for 6 hours at 37°C with virus peptide pools at final concentrations of 2 μg/mL of each peptide in the presence of CD28 and CD49d (BD Biosciences, Franklin Lakes, NJ) (Supplementary Table 3). After the stained cells were washed, flow cytometry data were collected on an LSRII Fortessa instrument (BD Biosciences). Compensation was performed using tubes of CompBeads (BD Biosciences) individually stained with each fluorophore, and compensation matrices were calculated with FACSdiva. Data were analyzed using FlowJo software, version 9 (Tree Star, Ashland, OR). Gating strategy and an example of raw flow cytometry data are shown in Supplementary Figure 1. A boolean gate platform was used with individual cytokine gates to create all possible response pattern combinations (Supplementary Data).

## RESULTS

### Baseline Characteristics

Between July 13, 2016, and September 19, 2017, 59 subjects were enrolled: 45 cases and 14 controls (Supplementary Figure 2). Approximately two-thirds were women in both the case and control groups (Table 1). Hispanic ethnicity was more prevalent in the ZIKV cases compared with controls (29% vs 7%, respectively), as was serologic evidence of prior DENV exposure (29% vs 14%, respectively) (Table 1). The median follow-up duration was 275 days.

**Table 1.** Baseline Characteristics of Subjects With Zika Infection (Cases) and Test-Negative Controls^a^

	Cases (n = 45)	Controls (n = 14)
Female, No. (%)	31 (68.9)	9 (64.3)
Ethnicity, No. (%)		
Hispanic	13 (28.9)	1 (7.1)
Non-Hispanic	32 (71.1)	13 (92.9)
Race, No. (%)		
Black/African American	3 (6.7)	T0
White	33 (73.3)	12 (85.7)
Multiracial	4 (8.9)	2 (14.3)
Unknown	5 (11.1)	0
Median age (range), y	44 (18 to 68)	31 (25 to 61)
Median enrollment DPO (range)^b^	72 (6 to 174)	15 (–3 to 357)
Flavivirus vaccination, No. (%)		
Yellow fever vaccine	12 (26.7)	8 (57.1)
Japanese encephalitis vaccine	1 (2.2)	0
Tick-borne encephalitis vaccine	1 (2.2)	0
ZIKV exposure, No. (%)		
Transmission in the United States	2 (4.4)	0
International travel	43 (96)	14 (100)
Sexual contact	17 (37.8)	8 (57.1)
Serologic evidence of prior DENV infection, No. (%)^a^	13 (28.9)	2 (14.3)

Abbreviations: DENV, dengue virus; DPO, days post onset; ZIKV, Zika virus.

^a^Subjects with DENV1–4 NAb titers <250 were categorized as DENV-naïve; those with titers ≥250 against 1 or more DENV serotypes were categorized as DENV-experienced.

^b^For patients who were asymptomatic, we calculated the DPO based on timing of exposure (eg, travel) and assumed an incubation period of 7 days. The DPO was calculated by adding 7 days to the last day of exposure. For symptomatic patients, day 1 was the first day of symptoms, and enrollment DPO was calculated based on the onset of symptoms.

### Clinical Presentation

The ZIKV cases and controls were enrolled with a median DPO of 72 and 15 days, respectively. All ZIKV cases had at least 1 symptom. Maculopapular rash was the most commonly reported symptom in cases and controls (98% and 82%, respectively). Other common symptoms included fatigue (87% and 82%, respectively), arthralgia (82% and 54%, respectively), conjunctivitis (56% and 45%, respectively), headaches (53% and 73%, respectively), and myalgia (53% vs 72%, respectively). During a follow-up period of 26.9 person-years, no cases of GBS were detected. Among male patients (14 cases and 2 controls), genitourinary symptoms were uncommon. These included burning with urination in 2 cases and 1 control, burning with ejaculation (1 and 1, respectively), testicular pain (1 and 0, respectively), and scrotal redness (1 and 1, respectively). Among cases, fatigue was the symptom that lasted the longest, with a median duration of 16 days, followed by headaches (12 days), as well as arthralgia, joint swelling, and pain with eye movement (8 days for each) (Table 2).

**Table 2.** Symptom Severity and Duration Reported by Patients With Confirmed ZIKV Infection and Controls

Symptom	Cases (n = 45)	Controls (n = 11)	*P* Value^a^
Conjunctivitis			
Any (severe)	25 (2)	5 (0)	.738
Median duration (range), d	5 (2–45)	3 (2–10)	.064
Fatigue			
Any (severe)	39 (10)	9 (3)	.649
Median duration (range), d	16 (2–284)	8 (3–287)	.341
Fever			
Any (severe)	10 (3)	2 (2)	1.000
Median duration (range), d	3 (2–5)	6.5 (6–7)	.037
Headache			
Any (severe)	24 (3)	8 (1)	.319
Median duration (range), d	11.5 (3–307)	8.5 (3–123)	.499
Arthralgia			
Any (severe)	37 (12)	6 (1)	.104
Median duration (range), d	8 (3–130)	6.5 (3–123)	.673
Joint swelling			
Any (severe)	18 (7)	4 (0)	1.000
Median duration (range), d	8 (3–130)	11 (2–85)	.966
Maculopapular rash			
Any (severe)	44 (6)	9 (1)	.095
Median duration (range), d	5 (2–45)	6 (3–91)	.802
Myalgia			
Any (severe)	24 (5)	8 (0)	.319
Median duration (range), d	7.5 (2–36)	7 (3–85)	.930
Retro-orbital pain			
Any (severe)	19 (1)	3 (0)	.498
Median duration (range), d	7 (2–119)	3 (2–8)	.149
Pain with eye movement			
Any (severe)	15 (3)	1 (0)	.144
Median duration (range), d	8 (2–119)	12 (NA)	NA
Edema in extremities			
Any (severe)	21 (4)	3 (0)	.319
Median duration (range), d	6 (3–130)	16 (3–93)	.661

Abbreviation: ZIKV, Zika virus; d, days.

^a^The Fisher exact test was used for categorical variables, and the Wilcoxon rank test was used for duration of symptoms.

Of the 13 ZIKV cases who were DENV-experienced, 9 (69%) reported at least 1 severe symptom, 3 (23%) reported at least 1 moderate symptom, and 1 (8%) reported only mild symptoms. Among the 32 ZIKV cases who were DENV-naïve, the symptom severity distribution was 15 (47%), 14 (44%), and 3 (9%), respectively. The proportion of subjects reporting severe solicited symptoms in ZIKV cases was comparable to the proportion in controls.

### ZIKV RNA in Body Fluids Specimens

There were 116 serum samples and 117 samples each from whole blood, saliva, and urine tested from 45 ZIKV cases. The samples were collected between DPO 6 and 364. All specimens at all time points from controls tested negative for ZIKV RNA by PCR. When a series of samples of a given type were available from the same subject, a gradual decline in ZIKV RNA concentration was usually observed.

**Figure 1.**
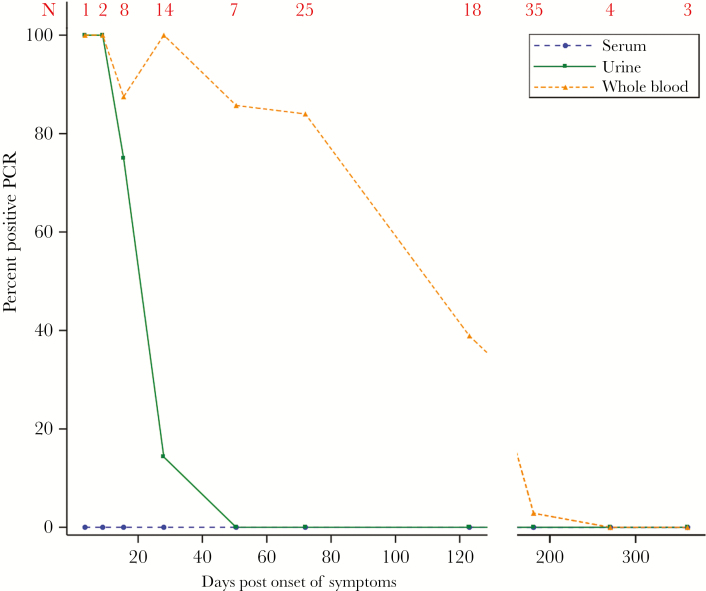
Zika virus (ZIKV) RNA persists much longer in whole blood than in either serum or urine. The 10 data points plotted for each of the 3 body fluid types shown here represent the proportions of each body fluid with positive ZIKV RNA detection within each time interval. The 10 time intervals were days post onset 1–6, 7–11, 12–19, 20–36, 37–64, 65–79, 80–166, 167–195, 196–345, and 346–375. The total numbers of whole blood, urine, and serum samples assessed during each interval are provided in red at the top of the figure.

Serum samples from ZIKV cases were negative at all time points. PCR sensitivity and the duration of positivity were highest for whole-blood specimens relative to serum and urine (Figure 1). The overall sensitivity was 94%: 51/57 (89%) of whole-blood samples were PCR-positive through DPO 79, 7/18 (39%) during DPO 80–166, and only 1/42 (2%) thereafter (Table 3). The latest positive whole-blood sample was collected on DPO 176.

**Table 3.** Whole-Blood PCR Sensitivity by DPO Window in Subjects With Confirmed ZIKV Infection (Cases)

DPO Window	No. Positive/Tested	Whole-Blood PCR Sensitivity^a^ in Window, %
DPO <7	1/1	100
DPO 7–11	2/2	100
DPO 12–19	7/8	88
DPO 20–36	14/14	100
DPO 37–64	6/7	86
DPO 65–79	21/25	84
DPO 80–166	7/18	39
DPO 167–195	1/35	3
DPO 196–34	0/4	0
DPO 346–375	0/3	0

Abbreviations: DPO, days post onset; PCR, polymerase chain reaction; ZIKV, Zika virus.

^a^The sensitivity of the assay was based on confirmed Zika virus infection, which was based on clinical presentation with history of exposure and the detection of ZIKV RNA in a body fluid sample, or positive ZIKV IgM and NAb in serum with low/no NAb against DENV1–4.

ZIKV RNA was also detected in urine by PCR. During the DPO 1–11 interval, 3/3 urine samples were PCR-positive, and 6/8 (75%) were positive during DPO 12–19. Detection in urine fell to 2/14 (14%) during DPO 20–36 and was negative at subsequent times.

The sole positive saliva sample of 117 tested was obtained at DPO 6. A total of 78 vaginal secretion samples from 31 ZIKV cases were collected from DPO 6–351. Three of these samples tested positive for ZIKV RNA, collected on DPO 6, 7, and 13 (the latter 2 from the same subject). Two of 21 semen samples from 10 cases obtained on DPO 14–364 were positive and were collected on DPO 26 and 34. The single breast milk sample obtained on DPO 99 tested negative for ZIKV RNA.

### Antibody Responses to ZIKV and DENV

**Figure 2.**
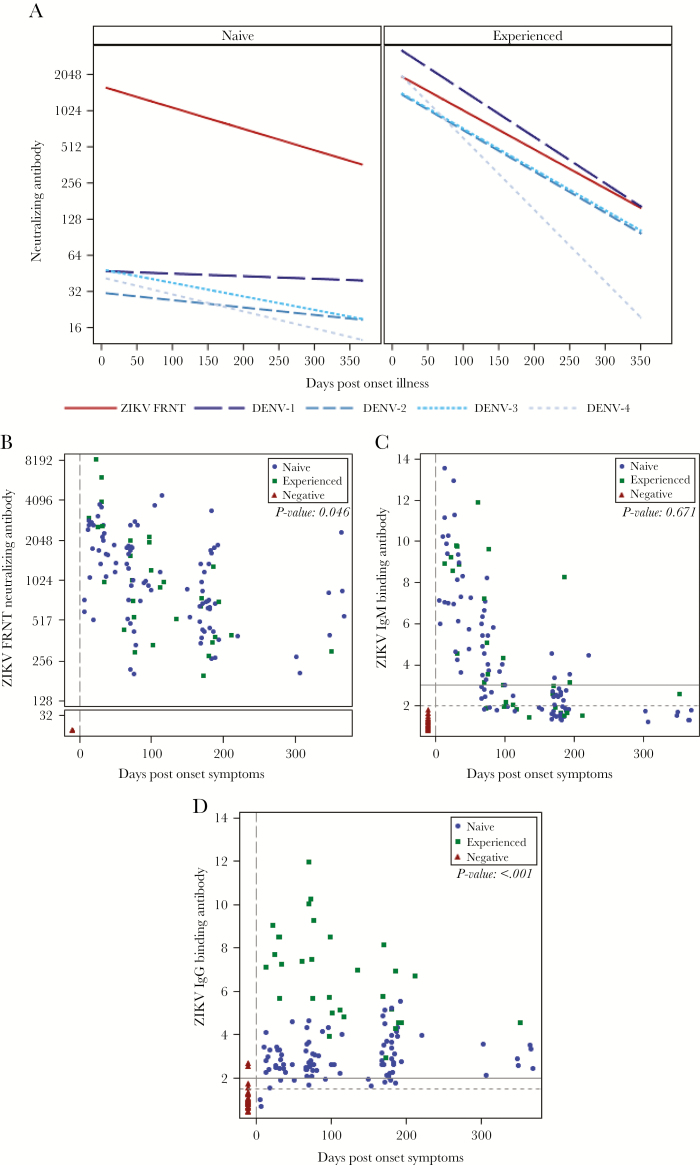
Serum-neutralizing antibody, immunoglobulin M (IgM), and IgG titers against Zika virus (ZIKV) and/or dengue virus (DENV) 1–4 over time in DENV-naïve patients, DENV-experienced patients, or controls. A, Best fit curves for focus reduction neutralization test (FRNT)_50_ titers against ZIKV and DENV1–4. The 45 patients with ZIKV infection were categorized as DENV-naïve (left panel) or DENV-experienced (right panel). Supplementary Table 4 provides the full antibody results for each time point for each subject and categorizes DENV-experienced vs -naïve cases and controls. B, All NAb time points for cases (DENV-experienced, green; DENV-naïve, blue) and controls (red). ZIKV-specific NAb titers (FRNT_50_) for all cases persisted with a half-life of 105 days. NAb titers were higher for DENV-experienced cases (*P* = .046), particularly early cases (ie, through day ~50). C, ZIKV cases anti-ZIKV IgM Ab levels were highest at the earliest time points available and decreased over time to negative (or equivocal) levels. IgM against ZIKV was not detected in controls. Solid horizontal line, positive cutoff (≥3); dashed horizontal line, equivocal cutoff (2–2.99). No significant difference in IgM levels was observed between DENV-experienced and -naïve patients (*P* = .671). D, Anti-ZIKV IgG levels were significantly higher in DENV-experienced patients relative to DENV-naïve (*P* < .001). Solid horizontal line, positive cutoff (≥2); dashed horizontal line, equivocal cutoff (1–1.99). One control had 2 low-level positive anti-ZIKV IgG values (control subject ZZ155 in Supplementary Table 4), likely due to cross-reactive IgG induced by a remote DENV infection. That subject also had low-level anti-DENV NAb as well.

DENV1–4 NAb titers were measured to determine the likelihood of past DENV infection (Figure 2A; Supplementary Table 4, Supplementary Figure 3). For persons with an initial serum sample collected after DPO 10, serum DENV Nab levels were generally highest in the initial sample and declined over time, suggesting that these represented cross-reactive or anamnestic (or both) responses triggered by ZIKV infections. These responses were significantly higher against DENV1 than the other DENV serotypes (*P* < .03) (Figure 2A).

ZIKV NAb were detectable in all ZIKV cases (n = 45), and in none of the 11 controls (Figure 2B). Through DPO 60, ZIKV NAb titers were higher in DENV-experienced subjects compared with DENV-naïve subjects (*P* = .027), and there was an overall difference between the 2 groups across all time points (*P* = .046) (Figure 2B). ZIKV NAb persisted through the latest available time point in all subjects, with a half-life of 105 days (95% confidence interval [CI], +/-17.69). The magnitude of ZIKV NAb titers was not associated with the duration of ZIKV RNA detection in whole blood (*P* = .084) (Supplementary Figure 4A) and was not significantly associated with self-reported prior receipt of yellow fever virus vaccine (*P* = .203) (Supplementary Figure 4B).

Anti-ZIKV IgM remained positive for a mean of ~4 months (DPO 124; 95% CI, +/-15 days) (Figure 2C). One case remained IgM-positive through ~7.5 months—the last time point assessed (DPO 220) (Figure 2C; subject ZZ138 in Supplementary Table 2). No difference in IgM was observed comparing the DENV-naïve and -experienced groups (*P* = .671) (Figure 2C). In contrast, anti-ZIKV IgG Ab levels peaked later than IgM and persisted through the latest assessments at ~1 year (Figure 2D); they were significantly higher in DENV-experienced subjects compared with DENV-naïve subjects (*P* < .001).

### ICS Results

**Figure 3.**
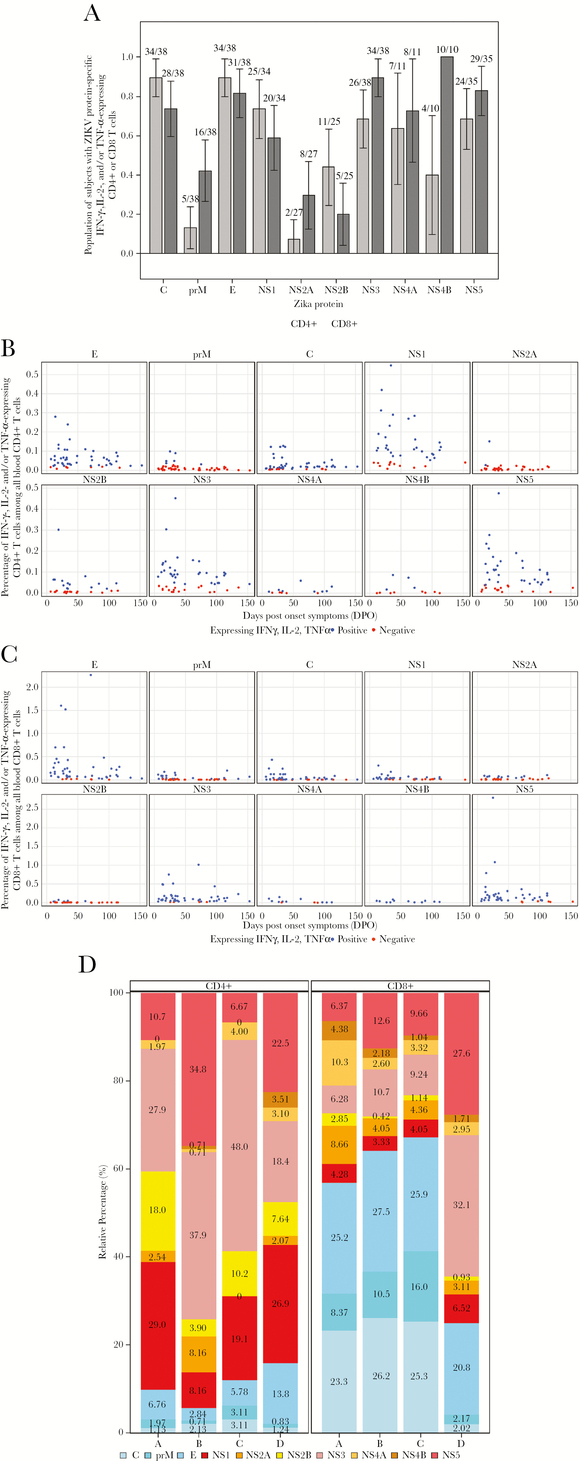
Zika virus (ZIKV)–specific CD4+ and CD8+ T cells producing interferon (IFN)-γ, interleukin (IL)-2, and/or tumor necrosis factor (TNF)–α cytokines after peptide stimulation in vitro. All 50 peripheral blood mononuclear cell (PBMC) samples (from 38 individuals) were tested against the structural proteins (E, C, and prM) and NS3; only 46, 42, 33, 31, 13, and 12 samples had sufficient PBMCs for testing against NS5, NS1, NS2a, NS2b, NS4a, and NS4B, respectively (Supplementary Data). A, Proportions of individuals with cytokine-expressing CD4+ T cells or CD8+ T cells against each ZIKV protein. PBMCs were analyzed in an intracellular cytokine staining assay for antigen-specific production of IFNγ, IL-2, and/or TNFα. Y-axis, proportion of subjects making a response against each of the 10 ZIKV proteins. The rank order for proportion of individuals with positive CD4+ T-cell responses was E, C, NS1, NS3, NS5, NS4A, NS2B, NS4B, prM, and NS2A, with percentages of tested subjects responding of 86%, 78%, 69%, 66%, 65%, 62%, 44%, 33%, 12%, and 6%, respectively. The rank order for proportion of individuals with positive CD8+ T-cell responses was NS4B, NS3, NS5, E, NS4A, C, NS1, prM, NS2A, and NS2B, with percentages of 100%, 92%, 87%, 86%, 77%, 76%, 67%, 48%, 33%, and 23%, respectively. B, Magnitudes for ZIKV protein–specific CD4+ T-cell or CD8+ T-cell responses. Total cytokine responses (IFN-γ, IL-2, and/or TNF-α expression) for each of the 10 ZIKV proteins over time are shown. Blue dots, positive responses; red dots, negative responses. X-axis, days post onset of symptoms (DPO). Y-axis, percent cytokine-producing cells among all blood CD4+ or CD8+ T cells. NS1, NS5, and NS3 (with geometric mean [GM] values of 0.14%, 0.10%, and 0.094% of total CD4+ T cells, respectively) had the highest magnitudes, compared with structural proteins E and C (GM values at 0.055% and 0.054%, respectively) and NS2B, NS4B, prM, and NS4A proteins (GM values at 0.047%, 0.040%, 0.025%, and 0.018%, respectively). CD8+ T-cell magnitudes were generally higher than for CD4+ T cells (note the y-axis scale differences). C and D, Relative magnitudes among total cytokine response of 4 subjects’ ZIKV-specific CD4+ T-cell and CD8+ T-cell responses against each of the 10 ZIKV proteins. For the 4 subjects whose samples were tested against all 10 ZIKV proteins, the relative proportions of their total response magnitude that were targeted to each individual structural (C, prM, E) or nonstructural (NS1, NS2A, NS2B, NS3, NS4A, NS4B, NS5) protein are shown. The time points analyzed for subjects A, B, C, and D were 16, 27, 39, and 62, respectively.

T-cell intracellular cytokine staining (ICS) results for 50 samples from 38 ZIKV cases collected at DPO 6–153 were available for this analysis (Supplementary Tables 5 and 6). Antigen-specific CD4+ T cells producing IFN-γ, IL-2, and/or TNF-α were observed against all 10 ZIKV proteins. Structural proteins E and C had the highest proportions of positive responders (89%) (Figure 3A), whereas only 2 of 27 ZIKV cases tested (7%) had CD4+ T cells that responded to NS2A peptides. The magnitudes of CD4+ T-cell responses were highest for the nonstructural proteins NS1, NS5, and NS3 (Figure 3B; Supplementary Table 5, Supplementary Figure 5). CD8+ T cells producing IFN-γ, IL-2, and /or TNF-α were also observed for all 10 ZIKV proteins, but with NS3, NS5, and NS4B having the highest proportions of positive responders (89%, 82%, and 100% respectively) (Figure 3A). The CD4+ and CD8+ response magnitudes to the 10 tested ZIKV proteins tended to parallel each other (Figure 3C; Supplementary Table 6, Supplementary Figure 5).

The relative distribution of the protein-specific response magnitudes was further demonstrated by examining the total T-cell cytokine responses for 4 subjects who had sufficient PBMCs for testing against the entire ZIKV proteome (Figure 3D). CD4+ T-cell response magnitudes were highest against nonstructural proteins (red color scheme), whereas CD8+ T cell magnitudes were highest against structural proteins (blue color scheme). We identified a strong polyfunctional response pattern for ZIKV-specific CD8+ T cells, with 69% and 62% of cells expressing 2–5 cytokines against NS5 and E proteins, respectively (Supplementary Figure 7). CD4+ T cells produced 2 or 3 cytokines in 27% and 53% of cells’ cytokines against NS5 and E, respectively.

## DISCUSSION

In this study of US patients with Zika infection, we found that symptoms and symptom severity were mostly comparable between 45 ZIKV cases and 14 controls, with the latter likely having an alternate undiagnosed viral infection. We found no specific sign or symptom that differentiated the cases and the controls. This self-limiting, nonspecific ZIKV disease pattern in adults makes for a diagnosis that may easily be missed outside of an epidemic setting. Seroprevalence studies demonstrated that 33%–80% of ZIKV infections are subclinical [4, 22].

We demonstrated that the sensitivity of detecting ZIKV by PCR is higher in whole-blood specimens compared with other body fluids in the early to late convalescent phases of the disease (up to 4 months). This is important information for epidemiologic surveillance and diagnostic purposes and has been suggested by smaller case series [23, 24]. Strikingly, the sensitivity of ZIKV PCR in whole blood remained >80% through DPO 79 before decreasing to 39% in the DPO 80–166 window. It is unknown if this prolonged detection reflects persistent replication and infectivity, as viral cultures were not incorporated into our study design.

Improved viral RNA detection by PCR for whole blood compared with serum was observed with other flaviruses [25]. Using seroconversion as the standard, Klungthong et al. found that the sensitivity of DENV RNA PCR was 74% in whole-blood samples compared with 52% in plasma/serum samples [26]. In a study from 20 blood donors with positive nucleic acid tests for West Nile virus (WNV), the viral load in the whole-blood red blood cell compartment exceeded that of plasma by 1 order of magnitude [27]. In 54 blood donors with positive WNV RNA PCR, 100% of plasma samples were PCR-negative at 3 weeks, whereas 42% of the whole-blood samples were still positive at 2 months [28]. Confirmation of our ZIKV findings in studies prospectively enrolling persons in the acute and convalescent phases of ZIKV infection, including those with subclinical infection, is an important future direction to advance ZIKV diagnostics and further our understanding of ZIKV transmission dynamics. Currently, diagnostic approaches are focused on PCR in serum specimens, which have a short window of detection of less than 8 days after symptom onset, but a recently approved test allows for testing of whole-blood specimens under the Food and Drug Administration Emergency Use Authorization, though not as a point of care (POC) test.

Upon categorizing the 45 ZIKV cases as DENV-experienced (n = 13) or -naïve (n = 32), we identified interesting serologic findings. Serum anti-ZIKV IgM positivity persisted until ~DPO 100 in most patients, although by ~DPO 200 a few sera remained positive and many were equivocal, consistent with a prior report [29, 30]. The magnitude and persistence of serum anti-ZIKV IgM were not diminished in DENV-experienced ZIKV cases, compared with DENV-naïve ones. This contrasted with reports for dengue infections in which anti-DENV IgM antibodies in secondary infections were reduced relative to primary infections, and with a recent report in which ZIKV IgM were reduced in Zika-infected, DENV-experienced patients [31–33]. Conversely, anti-ZIKV IgG was substantially higher in DENV-experienced ZIKV cases relative to dengue-naïve ones. We propose that a high level of anti-ZIKV IgG during ZIKV infection is a useful way to identify DENV-experienced patients (eg, an index >5 in our IgG assay). Similarly, in DENV-experienced ZIKV cases, anti-ZIKV NAb levels were significantly higher early (through DPO 60). A small series of acute Zika in DENV-experienced patients reported recall of DENV-specific memory B cells that produced antibody-secreting cells (ASCs) and NAb against DENV initially, and later ASC and NAb against ZIKV [34]. Hence anamnestic, cross-reactive responses are triggered by ZIKV infection in those with previous DENV infection, and that is another variable to consider in vaccine development planning in endemic regions.

ZIKV-specific antiviral T cells producing cytokines were detected by ICS for all 10 ZIKV proteins and were highly polyfunctional. The magnitudes of response were generally parallel between CD4+ and CD8+ T cells. Responses remained detectable in some patients for up to 10–12 months postinfection. In contrast to IgG and NAb, we did not identify a broad effect of prior DENV experience on T-cell responses, although CD8+ T cells’ magnitude of response against C, prM, and NS1 were higher for DENV-naïve patients, which is in line with findings by Grifoni et al. [35].

Our data need to be interpreted in the context of the study limitations. First, selection bias due to health care–seeking behavior resulted in enrollment of symptomatic patients. Our study cannot address asymptomatic infection or subclinical disease. Second, recall bias may have resulted from enrolling most of our patients in the convalescent phase of the disease. This may have impacted our analysis of the symptoms’ severity. As a corollary, enrolling most patients in the convalescent phase diminished our ability to compare the sensitivity of ZIKV RNA PCR assays in various body fluids during acute illness.

In conclusion, ZIKV RNA detection by PCR was much more prolonged in whole-blood samples compared with other body fluids. Development of diagnostic molecular assays on this easily obtained fluid should be prioritized for POC testing. Practical uses for a POC whole-blood PCR test include an improved molecular diagnostics window for recent (out to ~4 months) ZIKV infections, especially in DENV-endemic regions where ZIKV antibody diagnostics are difficult to interpret due to cross-reactive antibodies. This study detailed robust antibody and cellular responses during these natural infections and will provide important benchmarks for vaccine development efforts.

## Supplementary Material

ofy352_suppl_supplementary_materials

ofy352_suppl_supplementary_table_s4

ofy352_suppl_supplementary_table_s5

ofy352_suppl_supplementary_table_s6
