# Supplementary material for: Clinical, Virologic, and Immunologic Characteristics of Zika Virus Infection in a Cohort of US Patients: Prolonged RNA Detection in Whole Blood
Source: Open Forum Infect Dis. 2018 Dec 19;6(1):ofy352. doi: 10.1093/ofid/ofy352 (PMC6343961; doi:10.1093/ofid/ofy352)

**Supplemental Material**

**Prolonged Zika Virus RNA Detection in Whole Blood, Clinical Presentation, and Humoral and Cellular Immunity in a Cohort of US Zika Patients**

H El Sahly et al.

**Supplemental Material: Contents**

**Supplemental Text** – page 1

**Supplemental Figures** – page 5

**Supplemental Tables** – page 13

Note: supplemental tables S4, S5, and S6 are attached in separate documents.

**Supplemental Text**

**Supplemental Methods Text**

**Further details of viral stock production.** ZIKV was passaged in infecting Vero cells (ATCC; CRL-1586) at a multiplicity of infection of 0.05 in serum-free minimal essential medium (MEM; Life Technologies Gibco). After a 1 hour infection at 37 °C, MEM supplemented with 10% (vol/vol) fetal bovine serum (FBS) and 1% antibiotic/antimycotic (Corning MT30004CI) were added. Upon observation of severe cytopathic effect on day 3, supernatants and cells were collected and supplemented with an additional 10% (vol/vol) FBS before freezing at –80°C. Virus-containing supernatants/cells went through 3 freeze-thaw cycles and were then spun down at 930×*g* for 10 min at 4°C. Supernatants were stored in liquid nitrogen until use. DENV 1-4 viruses were also passaged as described above and virus-containing supernatants were collected on days 11, 8, 8, and 8 post-infection for DENV1, 2, 3 and 4, respectively.

**Additional methods for FRNT.** Serially diluted, heat-inactivated sera were incubated with a previously titrated amount (60–100 focus forming units) of ZIKV or DENV 1-4 for 1 hour at 37 °C. Vero cell monolayers in 96-well plates were infected with the mixture for 1 hour at 37 °C. An overlay containing 2% (wt/vol) methylcellulose (Sigma; M0512-2506) was added to the cells. After a 2-3 day incubation at 37 °C, the cells were washed with PBS1X and fixed with a 1:1 mixture of acetone and methanol. Foci were stained with a pan-flavivirus mouse monoclonal D1-4G2-4–15 (MAB10216, EMD Millipore) for 2 hour followed by HRP-linked anti-mouse IgG (Cell Signaling; 7076S) for 1 hour and developed using TrueBlue peroxidase substrate (KPL; 50–78-02). Foci were manually counted using the ImageJ counting software (NIH, Javascript) in duplicate wells per sample until the number of spots equaled the number of spots in the virus only control. Once the equivalent to the virus only control was reached in each sample, no further dilution wells were counted. The FRNT50 titer was calculated based on these counts using GraphPad Prism software. The titer was determined as an FRNT50, so 50% focus reduction.

**Details of ZIKV and DENV-2 peptides used in ICS assays.** Peptides of ZIKV structural (capsid [C], prM and envelope [E]) and non-structural proteins (NS1, NS2A, NS2B, NS3, NS4A, NS4B, and NS5) were synthesized by JPT (Berlin, Germany) and GenScript (Piscataway, NJ, USA) separately. Large proteins were split into 2 or 3 pools, as depicted in Table S1.

Peptide pools for DENV-2 (New Guinea C: GenPept: AAA4294) were provided by BEI Resources (<https://www.beiresources.org)> as 15- to 20-mers with 10 or 11 amino acid overlaps for the E protein (#NR-509), 13- to 19-mers with 10 amino acid overlap from NS3 (#NR-507) and 15- to 17-mers with 11 to 13 amino acid overlaps for NS5 proteins (#NR-2746). Large proteins were split into 2 or 3 pools, as depicted in Table S2.

**ICS assay.** Negative control samples were left unstimulated, and positive control samples were treated with *Staphylococcus* enterotoxin B (Sigma) at a final concentration of 1 μg/mL. The six-hour viral peptide stimulations included: incubation with peptides for 2 hours at 37°C, and then additional of a cocktail containing brefeldin A and monensin (eBioscience,004980-93), followed by additional 4 hours of culture. The cells were washed with 1xPBS; surface stained with Aqua live/dead stain L423102 (Biolegend), and then fixed and permeabilized using a Cytofix/Cytoperm kit (BD; 554722). The cells were then stained with fluorescence conjugated antibodies as listed in Table S3. Control PBMCs from 5 healthy subjects and 7 symptomatic subjects enrolled in the study but found to be ZIKV-uninfected were also assayed for responses to each of the ZIKV proteins to define the cut-off values for positive responses for each protein (geometric mean of the controls +3 SE). The earliest measurable ZIKV-specific T cell ICS responses against E or NS5 (as examples) were observed on DPOs 6-13 (see also Tables S5 and S6). The latest measurable T cell responses were observed on DPOs 104-153. Between 2-16 weeks post symptom onset, the total cytokine (CTK) response remained relatively high with the highest CD4+ response to E (0.28%) on DPO 14 and the highest CD8+ response to E (2.3%) on DPO 71. The highest CD4+ (0.47%) and CD8+ (2.8%) responses against NS5 were at DPOs 34 and 25, respectively.

**Details of Boolean analysis of ICS data.** Response to medium was subtracted from responses in stimulated samples for each of the response patterns. A minimum of 20 cytokine-positive cells were required for Boolean analysis. CD4+ and CD8+ T cells were then classified on the basis of IFN-γ, IL-2, TNF-α, CD107a and MIP-1-beta secretion as single, double, triple, quadruple and pentadruple producers (producing any 1, 2, 3, 4, and 5 cytokines respectively). The frequency of total antigen-specific CD4+ or CD8+ T cells was calculated by summing the frequency of CD4+ or CD8+T cells producing all not overlapping permutations of the cytokines tested.

**Supplemental Results Text**

**Impact of DENV-experience on CD8+ T Cell Response to ZIKV Infection.** In 13 subjects with visits between DPO 20-36, the GM of the CD8+ T cell response magnitudes were significantly higher in the DENV-naïve subjects (n=8) relative to the DENV-experienced subjects (n=5) for three ZIKV proteins: C, prM, and NS1 (P=0.02, 0.05, and 0.03, respectively; Table S9).

**Kinetics of the T Cell Response.** The kinetics of nine individual patients’ E- or NS5-specific CD4+ or CD8+ T cell responses are demonstrated in Figure S6. These were subjects for whom PBMCs from multiple time-points were available for this interim analysis.

**Supplemental Discussion Text**

**Categorization of Zika patients as DENV-experienced versus –naïve.** ZIKV infections in DENV-naïve subjects may stimulate low levels (e.g., titers <250) of de-novo cross-reactive NAb against DENV, whereas ZIKV infections in DENV-experienced subjects recall higher titers (>250) of NAb against DENV (Lai et al., 2018; Robbiani 2017). This cross-reactivity is due to amino acid homology (53.9-57.8%) between ZIKV and DENV-1-4 E proteins (Xu 2016), and recall of DENV-specific memory B cells by the incident ZIKV infection (Lai et al., 2018).

**IgM and IgG vs ZIKV.** In our study there was no detected impact of DENV-experience on anti-ZIKV IgM levels. This contrasts with secondary dengue infections where IgM titers were reported to be much lower than in primary dengue (Sa-Ngasang 2006, Changal 2016). This result suggests that with regard to IgM production, ZIKV is not behaving as a “fifth DENV serotype” as is sometimes suggested. In contrast, ZIKV-specific IgG responses were significantly higher in DENV-experienced subjects compared to naïve subjects and may be a useful tool in defining past DENV exposure (also suggested by Lanciotti, 2008).

**FRNT vs ZIKV.** In this study, during the first two months after symptom onset, ZIKV NAb were significantly higher in DENV-experienced subjects relative to naïve subjects. This was likely due to: 1) an anamnestic response in memory B cells (MBC) induced by previous dengue that are recalled, become plasmablasts, and produce NAb cross-reactive for ZIKV epitopes (Lai 2018); and 2) *de novo* activation of ZIKV-specific naïve B cells resulting in plasmablasts and NAb against type-specific ZIKV epitopes. DENV-naïve patients with acute Zika also have *de novo* activation of ZIKV-specific naïve B cells resulting in plasmablasts and NAb against type-specific ZIKV epitopes, but their early sera have less total NAb activity since they are lacking the cross-reactive DENV-specific MBC anamnestic component.

DENV-1 NAb titers were significantly higher than the other 3 DENV serotypes following ZIKV infections. DENV-1 does not have a substantially higher homology to ZIKV (Xu 2016), but it has been reported that a group of highly potent neutralizing monoclonal antibodies against only the DENV-1 and ZIKV E proteins are produced after ZIKV infection (Robbiani 2017).

**CD8+ T Cells.** In contrast to a report for dengue patients where the peak CD8+ T cell response magnitudes were highest against non-structural proteins (Rivino, Laura et al., 2012), in our Zika cohort the peak magnitudes for CD8+ T cell responses against the structural protein E (GM 0.156% of total CD8+ T cells) were similar to NS5 and higher than NS3 (GM values 0.155 and 0.103%, respectively). (Figures 3B and S6; Table S7).

**Supplemental Figures**

**Figure S1. Gating Strategy for Flow Cytometry and Cytokine Response after a ZIKV peptide pool stimulation.** (A). Successive gates were applied to identify singlet cells, lymphocytes and exclude dead cells, then identify CD3+ T cells and subsequently CD4+ or CD8+ T cells. (B) Antigen specificity of CD4+ and CD8+ T cells in a representative Zika patient. Flow cytometry plots showed IFN-γ, IL-2, TNF-α, CD107a and MIP-1α expression in response to stimulation with the peptide pool-1 from ZIKV E protein, in comparison to mock

stimulation.

**A.**

**B.**

CD4

CD8

Mock

ZIKV

E protein

Pool 1

Mock

IFN-γ

TNF-α

MIP-1β

IL-2

CD107a

ZIKV

E protein

Pool 1

0.002

0.006

0.013

0.002

0.001

0.173

0.065

0.2145

0.084

0.061

0.003

0.001

0.001

0.020

0.025

0.243

0.008

0.011

0.287

0.237

**Figure S2. Consort Flow Diagram.** Acute confirmed cases were those enrolled in the first 10 days after symptom onset; convalescent confirmed cases were those enrolled after that.


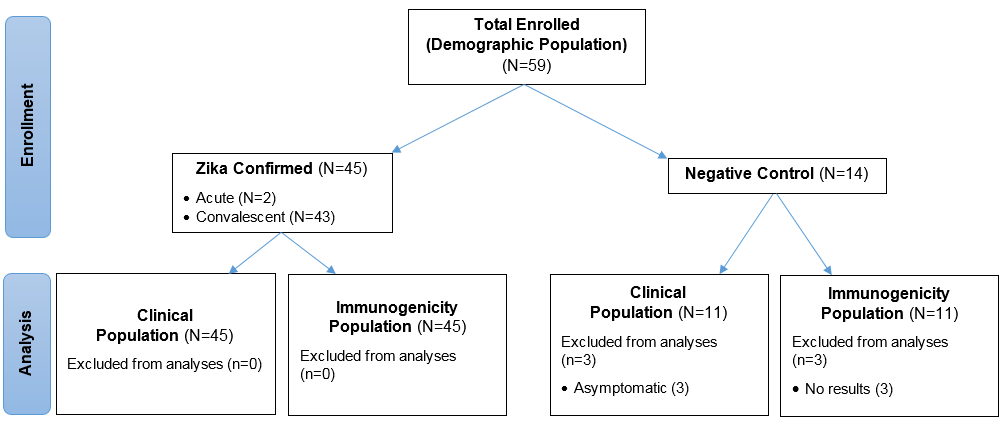


**Figure S3. Serum neutralizing antibody titers against DENV-1, DENV-2, DENV-3, or DENV-4 over time in DENV-naïve patients, DENV-experienced patients, or test-negative controls.**

**B)**

**A)**


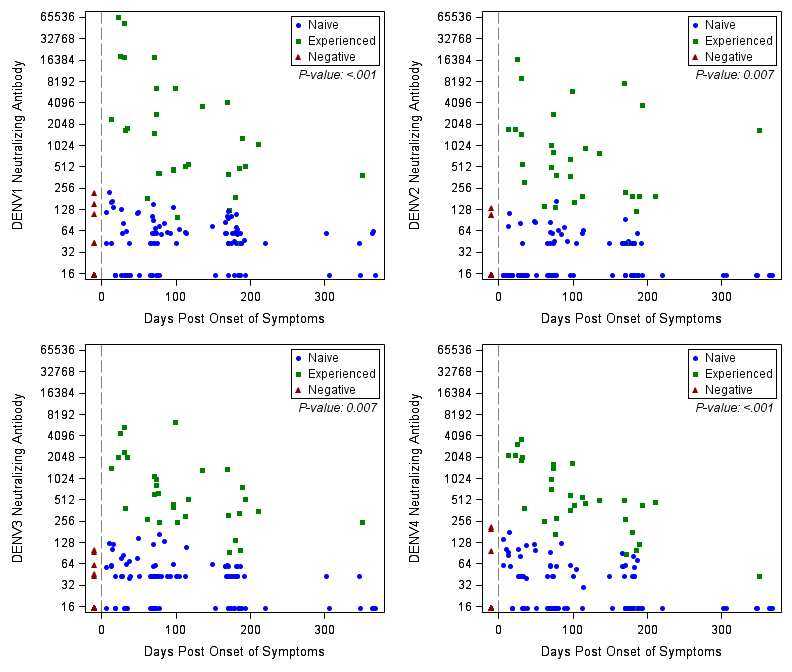


**C)**

**D)**

**Figure S4.** A. Magnitude of NAb response did not influence duration of ZIKV RNA persistence in whole blood ((p=0.084). B. Anti-ZIKV NAb titer magnitudes were not influenced by self-reported history of prior YFV vaccination.

**A.**


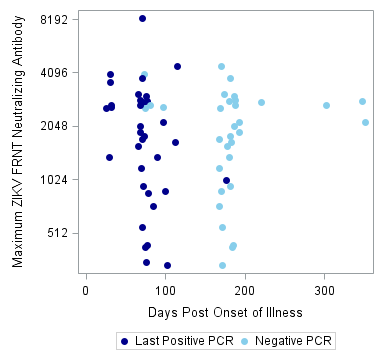


**B.**


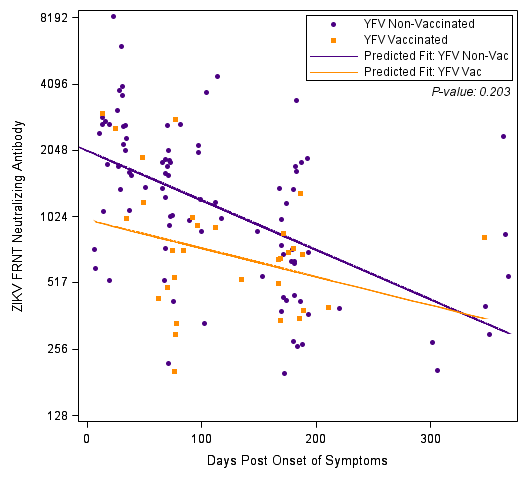


### Figure S5. Maximum Magnitudes of ZIKV Protein-specific CD4+ T Cell or CD8+ T Cell Responses (in ICS Assay; Production of IFN-γ, IL-2, and/or TNF-α at DPOs 6-153). The fractions above each bar indicate the numbers of subjects with a positive response over the numbers of subjects tested.


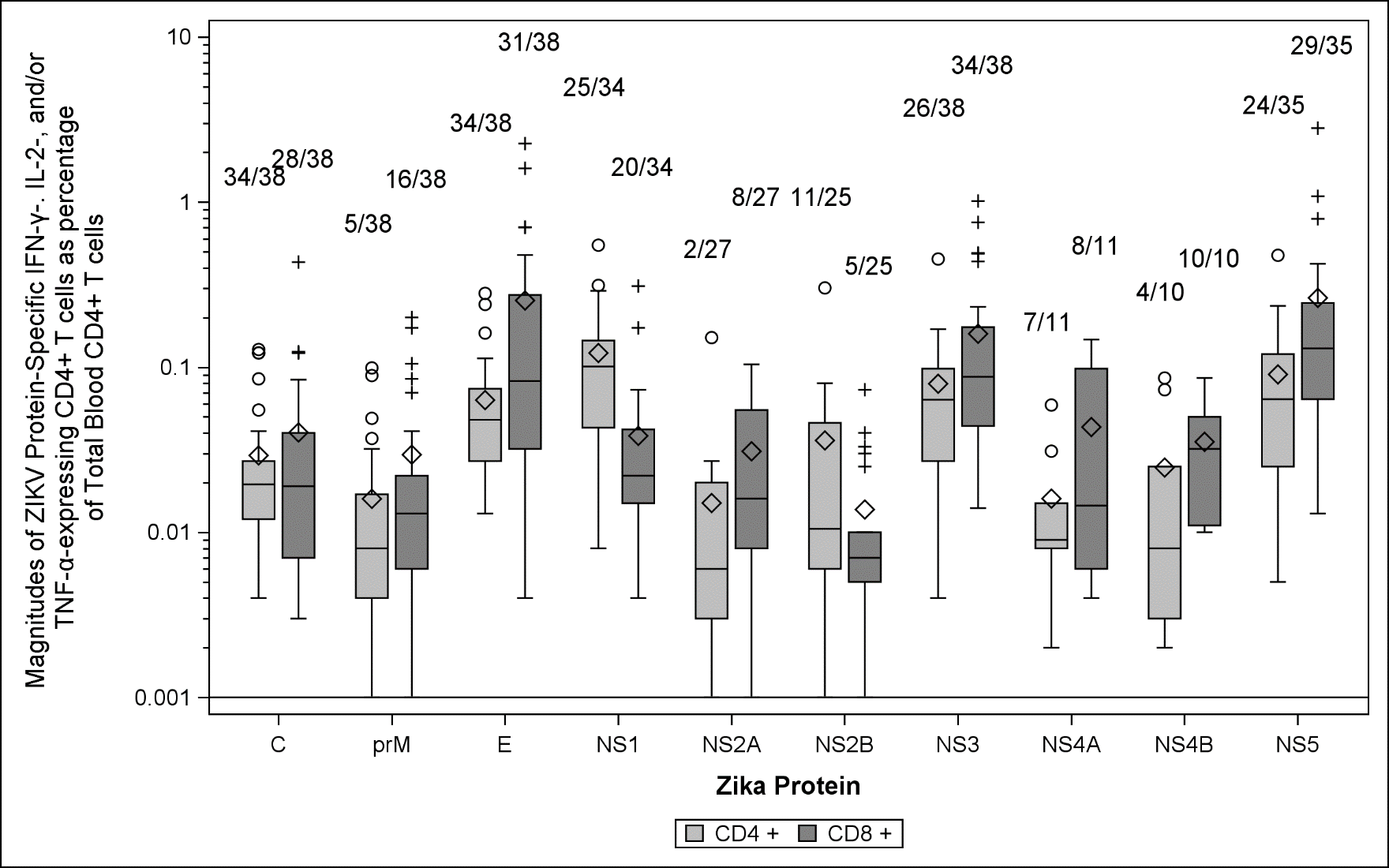


**Figure S6. Kinetics of nine individual patients’ E-specific or NS5-specific CD4+ or CD8+ T cell responses.** For nine individuals with PBMCs available from multiple time-points (DPO 6-34), the magnitudes of IFN-γ-, IL-2- and/or TNF-α-producing CD4+ or CD8+ T cells against either E and NS5 generally peaked prior to 3 weeks after symptom onset (DPO 21), or plateaued at all time points measured. A few patients had further increases in response magnitudes after three weeks. One example: the frequencies of all E- and NS5-specific T cell responses in subject ZZ128 (see gold dashed line with open triangles), a DENV-naïve subject, were higher at DPO34, the latest time point tested, for both CD4+ and CD8+ T cells (Tables S5 and S6). Also see Table S8.


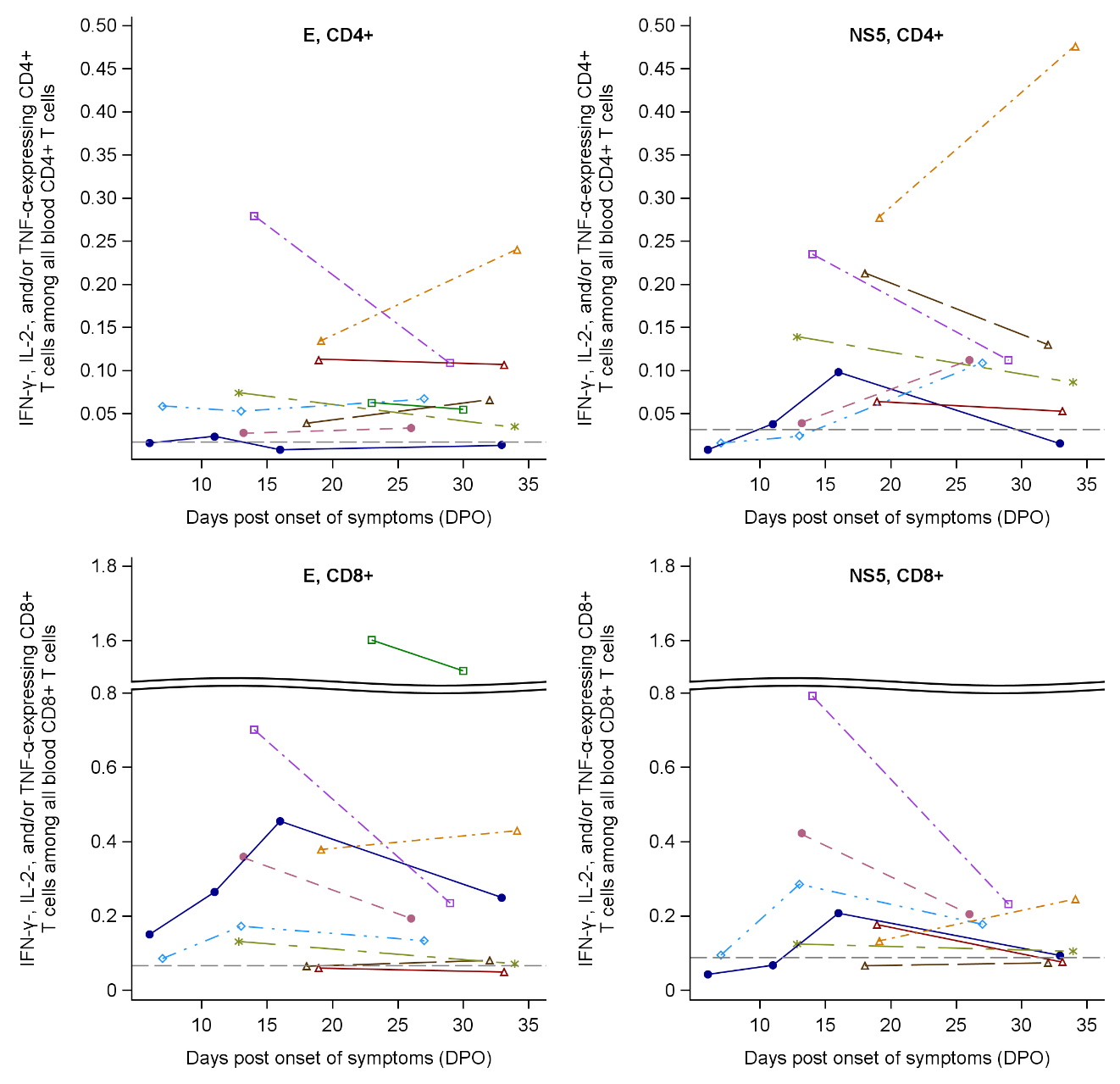


**Figure S7. High polyfunctionality of ZIKV E-specific or NS5-specific CD8+ and CD4+T cells.** The top two pie charts display the relative proportions of individual CD4+ T cells that produced 1, 2, 3, 4 and/or 5 cytokines (CTK). The bottom two pie charts display the relative proportions of individual CD8+T cells. The CTKs evaluated in ICS assays were IFNγ, IL-2, TNFα, MIP-1β and CD107a. We used a Boolean analysis against the ZIKV E (the two pie charts on the left) or NS5 (right pie charts) proteins. 5 CTK (purple), 4 CTK (brown), 3 CTK (orange), 2 CTK (green), or 1 CTK (blue); yellow is labeled “other” and indicates a low percentage of CD4+ T cells producing 4-5 cytokines. These nine subjects with samples from DPO 12-19 had strong polyfunctional response patterns for ZIKV-specific CD8+ T cells with 62% or 69% of cells expressing 2-5 cytokines against E or NS5 proteins, respectively. 53% and 27% of CD4+ T cells produced 2 or 3 cytokines against E and NS5, respectively.


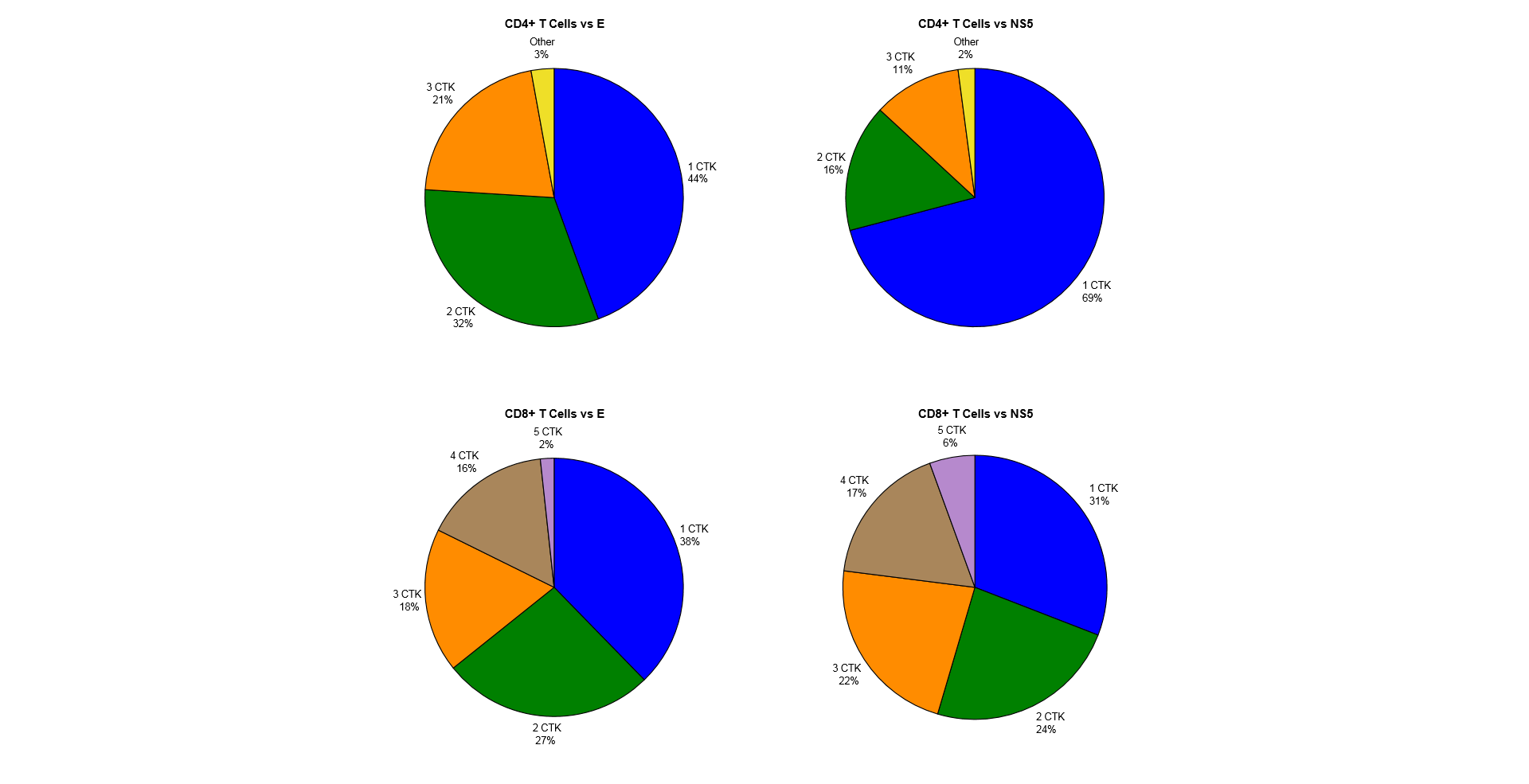


**Supplemental Tables**

**Table S1. Peptide pools for ZIKV-specific T cell assays**

| **Virus** | **Viral Protein** | **# Amino Acids** | **# Peptides** | **# pools** |
| --- | --- | --- | --- | --- |
| ZIKV | Capsid | 122 | 28 | 1 |
|  | prM | 168 | 40 | 1 |
|  | Envelop | 504 | 124 | 2 |
|  | NS1 | 352 | 85 | 2 |
|  | NS2a | 150 | 35 | 1 |
|  | NS2b | 126 | 29 | 1 |
|  | NS3 | 604 | 133 | 2 |
|  | NS4a | 145 | 34 | 1 |
|  | NS4b | 239 | 41 | 1 |
|  | NS5 | 639 | 157 | 3 |

**Table S2. Peptide pools for DENV-2-specific T cell assays**

| **Virus** | **Viral Protein** | **# Amino Acids** | **# Peptides** | **# pools** |
| --- | --- | --- | --- | --- |
| DENV-2 | Envelope | 495 | 67 | 1 |
|  | NS3 | 617 | 83 | 1 |
|  | NS5 | 820 | 156 | 2 |

**Table S3. Fluorescence Conjugated Antibodies Used in ICS Assay**

|  | Antibody | Clone | Vendor | Cat # |
| --- | --- | --- | --- | --- |
|  | CD3 | SP34-2 | BD Biosciences | 562877 |
|  | CD4 | L200 | BD Biosciences | 560836 |
|  | CD8 | RPA-T8 | BD Biosciences | 555367 |
|  | IFN- γ | 4S.B3 | eBioscience | 47731941 |
|  | IL-2 | MQ1-17H12 | BD Biosciences | 554567 |
|  | TNF- α | MAB11 | BD Biosciences | 560679 |
|  | CD107a | H4.A3 | BD Biosciences | 555800 |
|  | MIP-1-ß | D231-1351 | BD Biosciences | 560687 |

**Table S4. Antibody Responses by subject and days post onset of symptoms (DPO) (see separate file)**

**Table S5: CD4+ T cell responses (see separate file)**

**Table S6: CD8+ T cell responses (see separate file)**

**Table S7. Proportions of Assays with Positive Responses, and Response Magnitudes, in ZIKV protein-specific CD4+ T Cell or CD8+ T Cell Assays** (production of IFNγ, IL-2 and/or TNFα in response to peptide pools in the ICS assay)

| **ZIKV protein** | **E** | **C** | **prM** | **NS3** | **NS5** | **NS1** | **NS2A** | **NS2B** | **NS4A** | **NS4B** |
| --- | --- | --- | --- | --- | --- | --- | --- | --- | --- | --- |
| **# samples tested** | 50 | 50 | 50 | 50 | 46 | 42 | 33 | 31 | 13 | 12 |
| **#+CD4+**  **(%)** | 43  (86) | 39  (78) | 6  (12) | 33  (66) | 30  (65) | 29  (69) | 2  (6) | 14  (45) | 8  (62) | 4  (33) |
| **#+CD8+**  **(%)** | 43  (86) | 38  (76) | 23  (48) | 46  (92) | 40  (87) | 28  (67) | 11  (33) | 7  (23) | 10  (77) | 12  (100) |
| **GM CD4+**  **(95% CI)** | 0.055  (0.046-0.067) | 0.025  (0.019-0.032) | 0.054  (0.033-0.087) | 0.094  (0.078-0.114) | 0.101  (0.080-0.126) | 0.140  (0.109-0.170) | 0.064  (?) | 0.047  (0.031-0.070) | 0.018  (0.008-0.040) | 0.040  (0.011-0.143) |
| **GM CD8+**  **(95% CI)** | 0.156  (0.112-0.218) | 0.032  (0.022-0.046) | 0.045  (0.032-0.064) | 0.103  (0.079-0.134) | 0.155  (0.118-0.203) | 0.040  (0.030-0.053) | 0.067  (0.056-0.082) | 0.033  (0.019-0.056) | 0.030  (0.014-0.067) | 0.026  (0.016-0.042) |

GM, geometric mean of the peak response magnitude detected.

**Table S8. Overview of kinetics of cytokine-expressing T cells**

|  |  | Earliest DPO* with Positive CTK Response | Latest DPO ** with Positive CTK Response | DPO window with CTK magnitude >0.1% present | DPO when highest  CTK magnitude detected  (%CTK+ among total blood CD4+ or CD8+ T Cells) |
| --- | --- | --- | --- | --- | --- |
| CD4 | E | 7 | 153 | 14-75 | 14 (0.28) |
|  | NS5 | 13 | 153 | 13-112 | 34 (0.47) |
| CD8 | E | 6 | 153 | 13-117 | 71(2.26) |
|  | NS5 | 6 | 104 | 13-112 | 25 (2.8) |

*Earliest sample available was DPO 6.

**Latest sample available was DPO 153.

CTK, ZIKV Protein-Specific IFN-γ-, IL-2- and/or TNF-α-expressing CD4+ or CD8+ T Cells

**Table S9** To analyze the influence of previous DENV experience on the kinetics and magnitudes of the ZIKV-specific T cell responses against 3 structural and 3 nonstructural proteins, we further evaluated 13 subjects with visits between DPO 20-36. Five were DENV-experienced and eight were DENV-naïve. The GM of the CD8+ T cell response magnitudes were significantly higher in the DENV-naïve subjects relative to the DENV-experienced subjects for three ZIKV proteins: C, prM, and NS1. Overall, there was a trend for higher response magnitudes in naïve subjects, but the small numbers available for this analysis limited power to draw definitive conclusions.


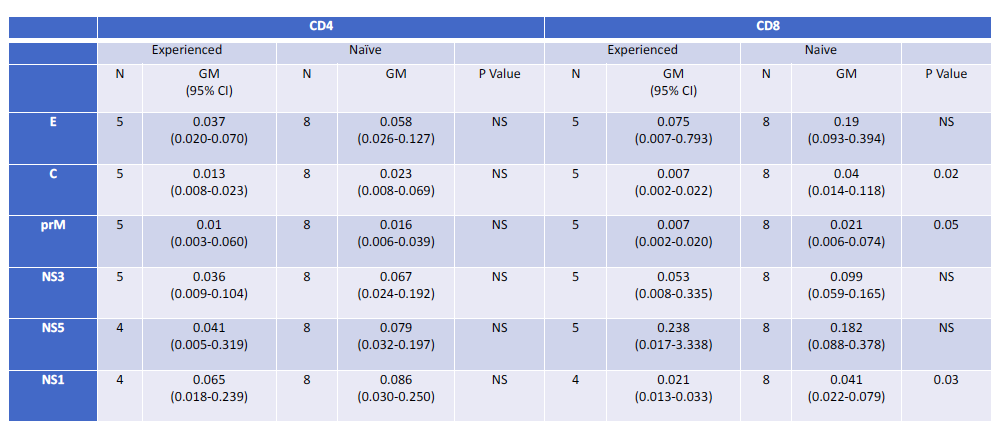

Supplement: ofy352_suppl_supplementary_materials [file ofy352_suppl_supplementary_materials.docx]
