# Supplementary material for: Clinical, Virologic, and Immunologic Characteristics of Zika Virus Infection in a Cohort of US Patients: Prolonged RNA Detection in Whole Blood
Source: Open Forum Infect Dis. 2018 Dec 19;6(1):ofy352. doi: 10.1093/ofid/ofy352 (PMC6343961; doi:10.1093/ofid/ofy352)
Supplement: ofy352_suppl_supplementary_table_s4 [file ofy352_suppl_supplementary_table_s4.docx]

**Table S4 Antibody Responses.** The 45 ZIKV-infected study participants (cases) were

characterized as DENV-experienced (n=13; shaded gray in the table) if the NAb

titer (FRNT) to DENV 1, 2, 3, and/or 4 was >250; or as DENV-naïve if <250 (n=32).

The last 11 subjects in the table were test negative controls whose diagnostic testing

did not confirm ZIKV infection.

| **ZIKA Confirmed?** | **Subject ID** | **Sex** | **DPO** | **ZIKV IgG** | **ZIKV IgM** | **ZIKV FRNT** | **DENV 1 FRNT** | **DENV 2 FRNT** | **DENV 3 FRNT** | **DENV 4 FRNT** |
| --- | --- | --- | --- | --- | --- | --- | --- | --- | --- | --- |
| Yes | ZZ124 | F | 71 | 1.66 | 2.82 | 221.5 | 42.4 | 15 | 15 | 15 |
|  |  |  | 171 | 1.81 | 1.52 | 441.4 | 42.4 | 15 | 42.4 | 15 |
|  |  |  | 368 | 2.43 | 1.78 | 550 | 15 | 15 | 15 | 15 |
| Yes | ZZ138 | F | 6 | 1 | 7.14 | 726.8 | 42.4 | 15 | 57.2 | 60.5 |
|  |  |  | 11 | 3.4 | 10.22 | 2450 | 224.2 | 15 | 123.4 | 102 |
|  |  |  | 16 | 2.99 | 9.87 | 2779 | 136.8 | 15 | 119.7 | 59.1 |
|  |  |  | 33 | 2.82 | 9.38 | 2651 | 62.3 | 15 | 62.9 | 42.4 |
|  |  |  | 72 | 2.61 | 7.08 | 1845 | 69 | 15 | 42.4 | 15 |
|  |  |  | 220 | 3.94 | 4.48 | 391.3 | 42.4 | 15 | 15 | 15 |
| Yes | ZZ114 | F | 72 | 2.5 | 2.44 | 932.9 | 60.7 | 59.1 | 60.3 | 42.4 |
|  |  |  | 181 | 3.97 | 1.35 | 642 | 42.4 | 42.4 | 42.4 | 15 |
| Yes | ZZ112 | M | 75 | 2.36 | 5.43 | 422.2 | 42.4 | 46 | 42.4 | 15 |
|  |  |  | 184 | 3.09 | 2.29 | 265 | 15 | 15 | 15 | 15 |
| Yes | ZZ108 | F | 37 | 2.41 | 7.3 | 1623 | 15 | 15 | 69.5 | 39.5 |
|  |  |  | 68 | 3.55 | 4.88 | 1253 | 59.4 | 15 | 15 | 15 |
|  |  |  | 192 | 5.55 | 1.84 | 1891 | 47.3 | 42.4 | 42.4 | 15 |
| Yes | ZZ136 | F | 19 | 1.51 | 7.02 | 523.4 | 15 | 15 | 42.4 | 15 |
|  |  |  | 33 | 1.88 | 6.12 | 2036 | 15 | 15 | 15 | 15 |
|  |  |  | 68 | 2.09 | 3.5 | 1864 | 15 | 15 | 15 | 15 |
|  |  |  | 186 | 1.74 | 1.47 | 423 | 15 | 15 | 15 | 15 |
| Yes | ZZ149 | F | 174 | 2.13 | 1.5 | 1178 | 103.7 | 46 | 42.4 | 15 |
| Yes | ZZ133 | M | 153 | 1.63 | 1.83 | 548.3 | 15 | 15 | 15 | 15 |
|  |  |  | 179 | 1.89 | 1.55 | 725.7 | 46 | 15 | 15 | 15 |
|  |  |  | 306 | 2.13 | 1.24 | 206 | 15 | 15 | 15 | 15 |
| Yes | ZZ127 | F | 117 | 4.84 | 1.83 | 1001 | 562.7 | 945.3 | 524.6 | 456.9 |
|  |  |  | 180 | 5.18 | 1.66 | 279 | 187.8 | 195 | 137.2 | 178 |
| Yes | ZZ104 | F | 135 | 7 | 1.44 | 529.2 | 3668 | 800.5 | 1336 | 508.1 |
|  |  |  | 211 | 6.71 | 1.51 | 397 | 1075 | 197 | 350 | 468 |
| Yes | ZZ129 | F | 23 | 9.06 | 9.24 | 8307 | 65194 | 1711 | 2045 | 2199 |
|  |  |  | 30 | 8.53 | 9.79 | 6094 | 53391 | 1477 | 2376 | 1834 |
|  |  |  | 71 | 10.05 | 3.13 | 2040 | 17754 | 500 | 602.2 | 707.1 |
| Yes | ZZ130 | F | 31 | 8.49 | 4.55 | 3993 | 17745 | 9114 | 5451 | 3687 |
|  |  |  | 73 | 10.26 | 1.89 | 1031 | 6608 | 827.5 | 830.9 | 1622 |
| Yes | ZZ137 | F | 96 | 4.34 | 4.04 | 939 | 135.2 | 15 | 63.1 | 60.5 |
|  |  |  | 188 | 4.31 | 2.74 | 690 | 42.4 | 15 | 15 | 15 |
| Yes | ZZ111 | F | 89 | 4.14 | 1.79 | 984 | 61.3 | 71.9 | 42.4 | 15 |
|  |  |  | 180 | 3.65 | 1.4 | 1361 | 61.7 | 42.4 | 42.4 | 15 |
| Yes | ZZ131 | F | 48 | 2.63 | 4.75 | 1893 | 113 | 86.8 | 76 | 121.3 |
|  |  |  | 77 | 2.6 | 3.71 | 2818 | 73.6 | 165.1 | 165 | 58.7 |
|  |  |  | 180 | 2.86 | 2.67 | 732.5 | 58.6 | 15 | 42.4 | 42.4 |
|  |  |  | 347 | 2.9 | 1.51 | 824 | 42.4 | 15 | 42.4 | 15 |
| Yes | ZZ123 | F | 18 | 2.38 | 9.42 | 1767 | 15 | 15 | 15 | 15 |
|  |  |  | 32 | 2.43 | 8.14 | 2171 | 15 | 42.4 | 15 | 15 |
|  |  |  | 81 | 2.33 | 2.67 | 2685 | 57.9 | 64.5 | 42.4 | 15 |
|  |  |  | 187 | 4.13 | 1.88 | 1803 | 59.7 | 59 | 58.8 | 71.9 |
|  |  |  | 302 | 3.55 | 1.74 | 275 | 59.5 | 15 | 42.4 | 15 |
| Yes | ZZ102 | F | 32 | 5.68 | 9.74 | 2628 | 1656 | 545.2 | 391.3 | 2013 |
|  |  |  | 97 | 3.91 | 3.02 | 1996 | 475.8 | 374.3 | 400.6 | 594.4 |
| Yes | ZZ118 | F | 37 | 2.59 | 3.63 | 1093 | 42.4 | 15 | 40 | 115.5 |
|  |  |  | 70 | 2.7 | 1.84 | 1726 | 59.7 | 42.4 | 42.4 | 59.2 |
|  |  |  | 168 | 3.15 | 1.36 | 1369 | 58.5 | 42.4 | 42.4 | 59.7 |
| Yes | ZZ125 | M | 49 | 4.58 | 5.74 | 1189 | 114.9 | 83 | 148.5 | 100.2 |
|  |  |  | 70 | 3.63 | 4.54 | 489.2 | 91.1 | 60.4 | 119.7 | 91.8 |
|  |  |  | 167 | 2.77 | 1.62 | 507.8 | 83.4 | 42.4 | 59.7 | 58.2 |
| Yes | ZZ122 | M | 25 | 7.7 | 8.57 | 2572 | 18241 | 16977 | 4378 | 3081 |
|  |  |  | 74 | 7.48 | 5.06 | 719.7 | 2769 | 2820 | 1002 | 1426 |
|  |  |  | 169 | 5.75 | 2.56 | 661.1 | 4106 | 7579 | 1360 | 495.1 |
| Yes | ZZ135 | F | 97 | 5.73 | 4.34 | 2163 | 461.4 | 652.8 | 436.7 | 359.9 |
|  |  |  | 193 | 4.53 | 3.15 | 707.1 | 510.9 | 3731 | 523.4 | 422.8 |
|  |  |  | 351 | 4.57 | 2.57 | 300 | 386 | 1664 | 244.3 | 42.4 |
| Yes | ZZ117 | M | 14 | 4.09 | 7.06 | 1076 | 167.3 | 111.4 | 101.6 | 178.6 |
|  |  |  | 29 | 3.27 | 4.62 | 1367 | 82.3 | 81.9 | 85.2 | 80.5 |
| Yes | ZZ126 | F | 73 | 2.79 | 3.29 | 1805 | 58.7 | 42.4 | 42.4 | 42.4 |
|  |  |  | 181 | 3.36 | 1.62 | 628.7 | 71 | 15 | 59.4 | 15 |
| Yes | ZZ115 | F | 62 | 7.37 | 11.9 | 436.5 | 182.2 | 142.5 | 274.6 | 257 |
|  |  |  | 77 | 9.28 | 9.61 | 297.8 | 415 | 385.3 | 244.1 | 276.4 |
|  |  |  | 185 | 6.92 | 8.25 | 354.8 | 489.3 | 118.6 | 327.5 | 98.7 |
| Yes | ZZ148 | M | 149 | 1.92 | 1.91 | 879.3 | 75 | 42.4 | 63.9 | 42.4 |
|  |  |  | 182 | 2.26 | 1.31 | 1723 | 67.3 | 42.4 | 59.7 | 57 |
|  |  |  | 364 | 3.53 | 1.29 | 2371 | 59.4 | 15 | 15 | 15 |
| Yes | ZZ101 | M | 28 | 3.41 | 11.27 | 3827 | 59.9 | 15 | 42.4 | 42.4 |
|  |  |  | 70 | 4.64 | 6.67 | 2650 | 150.3 | 84.9 | 59.8 | 60.4 |
|  |  |  | 181 | 5.21 | 2.52 | 450 | 109.6 | 15 | 42.4 | 82.3 |
| Yes | ZZ106 | M | 92 | 1.92 | 3.66 | 1015 | 58.1 | 45.6 | 42.4 | 15 |
|  |  |  | 176 | 2.03 | 2.47 | 705.1 | 59.7 | 42.4 | 42.4 | 15 |
| Yes | ZZ113 | F | 104 | 2.6 | 1.95 | 3742 | 67.2 | 42.4 | 42.4 | 53.1 |
|  |  |  | 183 | 2.63 | 1.49 | 3438 | 59.7 | 42.4 | 42.4 | 42.4 |
|  |  |  | 365 | 3.33 | 1.29 | 850 | 63.1 | 15 | 15 | 15 |
| Yes | ZZ153 | F | 51 | 1.88 | 7.28 | 1394 | 15 | 15 | 42.4 | 15 |
|  |  |  | 74 | 2.05 | 8.23 | 1033 | 15 | 15 | 15 | 15 |
|  |  |  | 193 | 2.74 | 3.54 | 366.7 | 15 | 15 | 15 | 15 |
| Yes | ZZ109 | M | 13 | 7.11 | 8.93 | 3028 | 2419 | 1709 | 1411 | 2191 |
|  |  |  | 34 | 7.23 | 8.89 | 1003 | 1791 | 306.1 | 2020 | 390.2 |
|  |  |  | 76 | 5.67 | 3.55 | 542.9 | 412.8 | 138.4 | 637.3 | 164.4 |
|  |  |  | 186 | 4.27 | 1.53 | 1308 | 42.4 | 15 | 99 | 15 |
| Yes | ZZ121 | M | 84 | 3 | 5.81 | 722.2 | 82.1 | 57.6 | 132 | 125 |
|  |  |  | 167 | 2.61 | 4.57 | 654.7 | 58.7 | 42.4 | 59.8 | 90.6 |
| Yes | ZZ132 | F | 7 | 0.69 | 6.01 | 597.1 | 117.5 | 15 | 15 | 142 |
|  |  |  | 13 | 2.81 | 11.14 | 2880 | 161.2 | 73.2 | 58.8 | 92.2 |
|  |  |  | 27 | 2.46 | 12.93 | 1733 | 129.7 | 15 | 77.1 | 101.9 |
|  |  |  | 68 | 2.4 | 6.34 | 734.5 | 103.4 | 15 | 42.4 | 15 |
|  |  |  | 188 | 3.9 | 1.73 | 268.3 | 42.4 | 15 | 15 | 15 |
| Yes | ZZ107 | F | 112 | 2.21 | 2.48 | 1193 | 60 | 58.8 | 42.4 | 15 |
|  |  |  | 183 | 2.72 | 1.95 | 1650 | 57.4 | 15 | 42.4 | 42.4 |
|  |  |  | 348 | 2.58 | 1.72 | 400 | 15 | 15 | 15 | 15 |
| Yes | ZZ120 | F | 114 | 3.99 | 1.75 | 4428 | 59.7 | 65.2 | 107.5 | 30 |
|  |  |  | 170 | 3.53 | 1.73 | 992.8 | 96 | 94.4 | 59.5 | 61.4 |
| Yes | ZZ110 | M | 13 | 2.27 | 13.58 | 2680 | 42.4 | 15 | 60 | 83.9 |
|  |  |  | 26 | 2.56 | 6.97 | 3093 | 15 | 15 | 42.4 | 42.4 |
|  |  |  | 66 | 2.46 | 2.93 | 1798 | 42.4 | 42.4 | 15 | 42.4 |
|  |  |  | 174 | 3.68 | 2.54 | 426 | 42.4 | 42.4 | 15 | 15 |
| Yes | ZZ105 | F | 39 | 2.27 | 8.31 | 1583 | 15 | 15 | 42.4 | 15 |
|  |  |  | 66 | 2.4 | 6 | 1374 | 15 | 15 | 42.4 | 15 |
|  |  |  | 178 | 2.22 | 2.83 | 641 | 15 | 15 | 15 | 15 |
| Yes | ZZ134 | F | 100 | 2.63 | 3.03 | 873.8 | 42.4 | 15 | 42.4 | 42.4 |
|  |  |  | 170 | 2.61 | 2.43 | 380 | 42.4 | 15 | 15 | 15 |
| Yes | ZZ119 | F | 112 | 5.15 | 2.06 | 916.4 | 518.4 | 198.3 | 297.9 | 555.8 |
|  |  |  | 189 | 4.56 | 1.64 | 385 | 1297 | 193 | 755.8 | 120 |
| Yes | ZZ103 | F | 71 | 11.97 | 7.24 | 1572 | 1503 | 1034 | 1107 | 980 |
|  |  |  | 170 | 8.14 | 2.99 | 760 | 395.8 | 225 | 308 | 275.4 |
| Yes | ZZ116 | M | 102 | 4.99 | 2.18 | 334.9 | 100.3 | 160.1 | 247.6 | 422.3 |
|  |  |  | 172 | 2.91 | 1.91 | 199 | 123 | 42.4 | 94.4 | 86.9 |
| Yes | ZZ128 | M | 19 | 3.3 | 10.27 | 2685 | 15 | 15 | 15 | 15 |
|  |  |  | 34 | 3.07 | 8.99 | 2320 | 15 | 15 | 15 | 15 |
|  |  |  | 68 | 4.31 | 5.44 | 1617 | 15 | 15 | 15 | 15 |
|  |  |  | 171 | 4.51 | 2.05 | 693 | 120 | 42.4 | 42.4 | 15 |
| Yes | ZZ152 | F | 31 | 3.3 | 4.26 | 3620 | 15 | 15 | 15 | 42.4 |
|  |  |  | 67 | 3.26 | 2.35 | 527.1 | 42.5 | 15 | 15 | 15 |
| Yes | ZZ144 | F | 78 | 3.07 | 1.92 | 333.6 | 15 | 15 | 15 | 15 |
|  |  |  | 171 | 5.14 | 3.36 | 860.4 | 15 | 15 | 15 | 15 |
| Yes | ZZ145 | M | 76 | 3.12 | 4.01 | 203.1 | 15 | 15 | 42.4 | 15 |
|  |  |  | 169 | 4.86 | 2.72 | 347.2 | 102.9 | 42.4 | 61 | 42.4 |
| Yes | ZZ147 | F | 99 | 8.51 | 1.97 | 1231 | 6473 | 5954 | 6281 | 1695 |
| No | ZZ156 | F | 63 | 0.47 | 1.48 | 15 | 15 | 15 | 15 | 15 |
|  |  |  | 78 | 0.4 | 0.87 | 15 | 42.4 | 15 | 15 | 15 |
|  |  |  | 183 | 1 | 1.09 | 15 | 15 | 15 | 15 | 15 |
| No | ZZ140 | F | 194 | 0.83 | 0.78 | 15 | 15 | 15 | 15 | 15 |
|  |  |  | 359 | 1.26 | 1.03 | 15 | 15 | 15 | 15 | 15 |
| No | ZZ155 | F | 40 | 2.66 | 0.85 | 15 | 109.6 | 104.9 | 61.9 | 209.9 |
|  |  |  | 67 | 2.54 | 0.89 | 15 | 213.7 | 131.8 | 93 | 198.9 |
|  |  |  | 171 | 1.71 | 1.17 | 15 | 151.2 | 106.4 | 99.5 | 15 |
| No | ZZ141 | M | 31 | 0.86 | 1.24 | 15 | 15 | 15 | 46 | 15 |
|  |  |  | 67 | 0.87 | 1.25 | 15 | 15 | 15 | 15 | 15 |
|  |  |  | 173 | 0.9 | 0.84 | 15 | 15 | 15 | 15 | 15 |
| No | ZZ150 | F | 6 | 0.65 | 1.27 | 15 | 15 | 15 | 15 | 15 |
|  |  |  | 11 | 1.22 | 1.79 | 15 | 15 | 15 | 15 | 15 |
|  |  |  | 19 | 0.82 | 1.34 | 15 | 15 | 15 | 15 | 15 |
|  |  |  | 33 | 0.6 | 1.24 | 15 | 15 | 15 | 15 | 15 |
|  |  |  | 65 | 0.69 | 1.27 | 15 | 15 | 15 | 15 | 15 |
| No | ZZ151 | F | 357 | 1.28 | 1.6 | 15 | 42.4 | 15 | 15 | 15 |
|  |  |  | 374 | 0.76 | 1.1 | 15 | 15 | 15 | 15 | 15 |
| No | ZZ142 | M | 7 | 0.83 | 1.36 | 15 | 15 | 15 | 15 | 15 |
|  |  |  | 9 | 0.89 | 1.25 | 15 | 15 | 15 | 15 | 15 |
|  |  |  | 21 | 1.05 | 1.08 | 15 | 15 | 15 | 15 | 15 |
| No | ZZ154 | M | 6 | 0.61 | 1 | 15 | 15 | 15 | 15 | 15 |
|  |  |  | 11 | 0.77 | 1.41 | 15 | 15 | 15 | 15 | 15 |
|  |  |  | 17 | 1.02 | 1.6 | 15 | 15 | 15 | 15 | 15 |
|  |  |  | 23 | 0.77 | 1.19 | 15 | 15 | 15 | 15 | 15 |
| No | ZZ143 | F | 72 | 1.53 | 1 | 15 | 15 | 15 | 42.5 | 95.7 |
| No | ZZ139 | F | 12 | 0.94 | 0.92 | 15 | 15 | 15 | 15 | 15 |
|  |  |  | 34 | 0.86 | 0.99 | 15 | 15 | 15 | 15 | 15 |
| No | ZZ146 | F | 14 | 0.83 | 1.03 | 15 | 15 | 15 | 15 | 15 |
|  |  |  | 32 | 0.9 | 0.96 | 15 | 15 | 15 | 15 | 15 |
|  |  |  | 73 | 0.98 | 1.06 | 15 | 15 | 15 | 15 | 15 |
|  |  |  | 183 | 1.32 | 0.87 | 15 | 15 | 15 | 15 | 15 |
