# Supplementary material for: Clinical, Virologic, and Immunologic Characteristics of Zika Virus Infection in a Cohort of US Patients: Prolonged RNA Detection in Whole Blood
Source: Open Forum Infect Dis. 2018 Dec 19;6(1):ofy352. doi: 10.1093/ofid/ofy352 (PMC6343961; doi:10.1093/ofid/ofy352)
Supplement: ofy352_suppl_supplementary_table_s5 [file ofy352_suppl_supplementary_table_s5.docx]

**Table S5** Frequencies of ZIKV-Specific CD4+ T Cells Producing IFN-γ, IL-2, and/or TNF-α

|  | | **ZIKV Proteins Tested (Peptide Pools)** | | | | | | | | | |
| --- | --- | --- | --- | --- | --- | --- | --- | --- | --- | --- | --- |
| **Subject ID** | **DPO** | **E** | **C** | **PRM** | **NS3** | **NS5** | **NS1** | **NS2A** | **NS2B** | **NS4A** | **NS4B** |
| ZZ124 | 71 | 0.016 | 0.006 | 0.002 | 0.027 | 0.008 | 0.013 | - | - | - | - |
| ZZ138 | 6 | 0.016 | 0.009 | 0.009 | 0.078 | 0.008 | 0.040 | 0.001 | 0.008 | - | - |
|  | 11 | 0.024 | 0.004 | 0.007 | 0.099 | 0.038 | 0.103 | 0.009 | 0.064 | 0.007 | 0.000 |
|  | 16 | 0.008 | 0.006 | 0.002 | 0.107 | 0.098 | 0.023 | 0.023 | 0.011 | 0.002 | 0.002 |
|  | 33 | 0.013 | 0.007 | 0.007 | 0.108 | 0.015 | 0.043 | 0.000 | 0.023 | 0.009 | 0.000 |
| ZZ114 | 72 | 0.072 | 0.035 | 0.011 | 0.152 | 0.154 | 0.284 | 0.005 | 0.027 | - | 0.025 |
| ZZ112 | 75 | 0.101 | 0.038 | 0.032 | 0.034 | 0.059 | 0.117 | 0.018 | - | - | - |
| ZZ108 | 37 | 0.161 | 0.122 | 0.022 | 0.089 | 0.024 | 0.074 | 0.008 | 0.007 | - | 0.086 |
| ZZ136 | 19 | 0.113 | 0.016 | 0.037 | 0.031 | 0.064 | - | - | - | - | - |
|  | 33 | 0.107 | 0.019 | 0.021 | 0.025 | 0.053 | 0.043 | 0.004 | 0.007 | - | - |
| ZZ133 | 153 | 0.026 | 0.020 | 0.000 | 0.043 | 0.025 | 0.041 | - | - | - | - |
| ZZ127 | 117 | 0.013 | 0.017 | 0.004 | 0.013 | 0.005 | - | - | - | - | - |
| ZZ104 | 135 | 0.024 | 0.018 | 0.001 | 0.026 | - | - | - | - | - | - |
| ZZ129 | 23 | 0.063 | 0.006 | 0.010 | 0.014 | - | - | - | - | - | - |
|  | 30 | 0.055 | 0.012 | 0.027 | 0.006 | - | - | - | - | - | - |
| ZZ130 | 31 | 0.017 | 0.027 | 0.006 | 0.015 | 0.015 | 0.029 | 0.007 | 0.000 | 0.002 | - |
| ZZ137 | 96 | 0.027 | 0.007 | 0.000 | 0.006 | - | - | - | - | - | - |
| ZZ111 | 89 | 0.019 | 0.021 | 0.002 | 0.004 | 0.008 | 0.022 | 0.001 | 0.002 | 0.000 | 0.002 |
| ZZ131 | 48 | 0.040 | 0.008 | 0.011 | 0.015 | 0.036 | 0.024 | 0.003 | 0.001 | - | - |
| ZZ123 | 18 | 0.039 | 0.085 | 0.020 | 0.151 | 0.213 | 0.313 | - | - | - | - |
|  | 32 | 0.066 | 0.067 | 0.049 | 0.088 | 0.130 | 0.232 | - | - | - | - |
| ZZ102 | 32 | 0.058 | 0.011 | 0.004 | 0.050 | 0.013 | 0.039 | 0.005 | 0.006 | - | - |
| ZZ118 | 37 | 0.033 | 0.024 | 0.017 | 0.170 | 0.152 | 0.290 | 0.025 | 0.041 | - | - |
| ZZ125 | 49 | 0.075 | 0.013 | 0.006 | 0.043 | 0.032 | 0.077 | 0.006 | 0.008 | - | - |
| ZZ122 | 25 | 0.037 | 0.012 | 0.099 | 0.121 | 0.173 | 0.173 | 0.003 | 0.058 | - | - |
| ZZ135 | 97 | 0.058 | 0.007 | 0.002 | 0.042 | 0.052 | 0.053 | 0.004 | 0.021 | 0.008 | - |
| ZZ117 | 14 | 0.280 | 0.122 | 0.022 | 0.144 | 0.235 | 0.190 | 0.008 | 0.007 | - | - |
|  | 29 | 0.108 | 0.122 | 0.022 | 0.081 | 0.112 | 0.108 | 0.008 | 0.007 | - | - |
| ZZ126 | 73 | 0.054 | 0.016 | 0.002 | 0.098 | 0.120 | 0.160 | 0.020 | - | - | - |
| ZZ115 | 62 | 0.110 | 0.020 | 0.007 | 0.157 | 0.191 | 0.269 | 0.024 | 0.080 | 0.059 | 0.073 |
| ZZ101 | 28 | 0.024 | 0.015 | 0.006 | 0.006 | 0.022 | 0.008 | 0.001 | 0.003 | 0.003 | - |
| ZZ106 | 92 | 0.066 | 0.020 | 0.011 | 0.048 | 0.065 | 0.068 | - | - | - | - |
| ZZ113 | 104 | 0.031 | 0.020 | 0.005 | 0.048 | 0.051 | 0.065 | 0.006 | 0.010 | 0.009 | 0.006 |
| ZZ109 | 13 | 0.074 | 0.004 | 0.001 | 0.136 | 0.139 | 0.116 | 0.027 | 0.064 | - | - |
|  | 34 | 0.034 | 0.013 | 0.007 | 0.075 | 0.086 | 0.093 | 0.005 | 0.021 | - | - |
| ZZ121 | 84 | 0.027 | 0.015 | 0.004 | 0.096 | 0.045 | 0.099 | 0.003 | 0.045 | 0.008 | 0.008 |
| ZZ132 | 7 | 0.059 | 0.010 | 0.005 | 0.034 | 0.016 | - | - | - | - | - |
|  | 13 | 0.053 | 0.010 | 0.006 | 0.016 | 0.024 | - | - | - | - | - |
|  | 27 | 0.067 | 0.006 | 0.004 | 0.089 | 0.109 | 0.130 | 0.010 | 0.037 | 0.015 | 0.017 |
| ZZ107 | 112 | 0.093 | 0.020 | 0.009 | 0.098 | 0.097 | 0.145 | 0.021 | 0.047 | 0.031 | 0.000 |
| ZZ120 | 114 | 0.074 | 0.025 | 0.014 | 0.092 | 0.064 | 0.121 | 0.016 | 0.011 | - | - |
| ZZ110 | 13 | 0.027 | 0.022 | 0.013 | 0.020 | 0.039 | 0.038 | 0.004 | 0.005 | - | - |
|  | 26 | 0.033 | 0.007 | 0.011 | 0.092 | 0.112 | 0.116 | - | - | - | - |
| ZZ105 | 39 | 0.029 | 0.034 | 0.002 | 0.008 | 0.005 | 0.033 | 0.001 | 0.006 | 0.000 | 0.003 |
| ZZ134 | 100 | 0.050 | 0.041 | 0.011 | 0.077 | 0.111 | 0.086 | - | - | - | - |
| ZZ119 | 112 | 0.046 | 0.055 | 0.017 | 0.084 | 0.089 | 0.105 | - | - | - | - |
| ZZ103 | 71 | 0.024 | 0.019 | 0.002 | 0.091 | 0.061 | 0.109 | 0.002 | 0.005 | - | - |
| ZZ116 | 102 | 0.036 | 0.004 | 0.006 | 0.048 | 0.041 | 0.082 | - | - | - | - |
| ZZ128 | 19 | 0.134 | 0.065 | 0.048 | 0.304 | 0.277 | 0.419 | 0.151 | 0.302 | - | - |
|  | 34 | 0.240 | 0.128 | 0.089 | 0.452 | 0.476 | 0.548 | 0.014 | 0.047 | - | - |
| **Cut off Value*** | **-** | **0.020** | **0.007** | **0.029** | **0.036** | **0.038** | **0.050** | **0.026** | **0.018** | **0.007** | **0.014** |
|  | | | | | | | | | | | |

***Cut-off for a positive response was defined as the geometric mean T cell response plus 3 SE of 5 healthy subjects and 7 subjects enrolled but**

**confirmed ZIKV-negative.**

**Cells with dashes in the table indicates that the peptide pool was not tested for that participant (due to limitations in PBMC availability).**

**DPO, days post onset of symptoms.**
