# Supplementary material for: Clinical, Virologic, and Immunologic Characteristics of Zika Virus Infection in a Cohort of US Patients: Prolonged RNA Detection in Whole Blood
Source: Open Forum Infect Dis. 2018 Dec 19;6(1):ofy352. doi: 10.1093/ofid/ofy352 (PMC6343961; doi:10.1093/ofid/ofy352)
Supplement: ofy352_suppl_supplementary_table_s6 [file ofy352_suppl_supplementary_table_s6.docx]

### Table S6 Frequencies of ZIKV-Specific CD8+ T Cells Producing IFN-γ, IL-2, and/or TNF-α

|  | | **ZIKV Proteins Tested (Peptide Pools)** | | | | | | | | | |
| --- | --- | --- | --- | --- | --- | --- | --- | --- | --- | --- | --- |
| **Subject ID** | **DPO** | **E** | **C** | **PRM** | **NS3** | **NS5** | **NS1** | **NS2A** | **NS2B** | **NS4A** | **NS4B** |
| ZZ124 | 71 | 0.014 | 0.020 | 0.006 | 0.056 | 0.024 | 0.004 | - | - | - | - |
| ZZ138 | 6 | 0.151 | 0.066 | 0.115 | 0.031 | 0.043 | 0.022 | 0.008 | 0.005 | - | - |
|  | 11 | 0.265 | 0.245 | 0.088 | 0.066 | 0.067 | 0.045 | 0.091 | 0.030 | 0.108 | 0.046 |
|  | 16 | 0.455 | 0.433 | 0.173 | 0.177 | 0.208 | 0.055 | 0.067 | 0.007 | 0.043 | 0.036 |
|  | 33 | 0.249 | 0.244 | 0.154 | 0.089 | 0.093 | 0.039 | 0.042 | 0.011 | 0.032 | 0.010 |
| ZZ114 | 72 | 0.009 | 0.030 | 0.005 | 0.014 | 0.013 | 0.005 | 0.001 | 0.001 | - | 0.010 |
| ZZ112 | 75 | 0.274 | 0.006 | 0.006 | 0.030 | 0.118 | 0.022 | 0.080 | - | - | - |
| ZZ108 | 37 | 0.072 | 0.122 | 0.022 | 0.175 | 0.130 | 0.010 | 0.008 | 0.007 | - | 0.086 |
| ZZ136 | 19 | 0.060 | 0.004 | 0.040 | 0.127 | 0.176 | - | - | - | - | - |
|  | 33 | 0.049 | 0.007 | 0.019 | 0.045 | 0.077 | 0.034 | 0.005 | 0.009 | - | - |
| ZZ133 | 153 | 0.028 | 0.010 | 0.070 | 0.035 | 0.029 | 0.006 | - | - | - | - |
| ZZ127 | 117 | 0.282 | 0.084 | 0.200 | 0.127 | 0.019 | - | - | - | - | - |
| ZZ104 | 135 | 0.045 | 0.003 | 0.020 | 0.232 | - | - | - | - | - | - |
| ZZ129 | 23 | 1.601 | 0.002 | 0.002 | 0.754 | - | - | - | - | - | - |
|  | 30 | 1.519 | 0.006 | 0.002 | 0.511 | - | - | - | - | - | - |
| ZZ130 | 31 | 0.020 | 0.030 | 0.010 | 0.018 | 0.074 | 0.018 | 0.015 | 0.005 | 0.005 | - |
| ZZ137 | 96 | 0.064 | 0.008 | 0.000 | 0.042 | - | - | - | - | - | - |
| ZZ111 | 89 | 0.027 | 0.003 | 0.001 | 0.036 | 0.074 | 0.015 | 0.013 | 0.003 | 0.000 | 0.050 |
| ZZ131 | 48 | 0.021 | 0.000 | 0.006 | 0.119 | 0.309 | 0.014 | 0.012 | 0.000 | - | - |
| ZZ123 | 18 | 0.065 | 0.042 | 0.010 | 0.053 | 0.066 | 0.019 | - | - | - | - |
|  | 32 | 0.081 | 0.046 | 0.022 | 0.058 | 0.074 | 0.021 | - | - | - | - |
| ZZ102 | 32 | 0.092 | 0.005 | 0.004 | 0.047 | 0.146 | 0.017 | 0.007 | 0.010 | - | - |
| ZZ118 | 37 | 0.179 | 0.008 | 0.013 | 0.055 | 0.099 | 0.038 | 0.012 | 0.010 | - | - |
| ZZ125 | 49 | 0.075 | 0.019 | 0.004 | 0.057 | 0.060 | 0.042 | 0.075 | 0.010 | - | - |
| ZZ122 | 25 | 0.012 | 0.003 | 0.012 | 0.015 | 2.806 | 0.019 | 0.009 | 0.001 | - | - |
| ZZ135 | 97 | 0.051 | 0.004 | 0.012 | 0.437 | 0.037 | 0.009 | 0.006 | 0.006 | 0.009 | - |
| ZZ117 | 14 | 0.702 | 0.122 | 0.022 | 0.487 | 0.792 | 0.310 | 0.008 | 0.007 | - | - |
|  | 29 | 0.235 | 0.122 | 0.022 | 0.117 | 0.232 | 0.098 | 0.008 | 0.007 | - | - |
| ZZ126 | 73 | 0.089 | 0.003 | 0.005 | 0.082 | 0.207 | 0.023 | 0.003 | - | - | - |
| ZZ115 | 62 | 0.065 | 0.024 | 0.040 | 0.093 | 0.132 | 0.073 | 0.044 | 0.040 | 0.098 | 0.039 |
| ZZ101 | 28 | 0.706 | 0.040 | 0.017 | 0.177 | 1.085 | 0.022 | 0.019 | 0.033 | 0.147 | - |
| ZZ106 | 92 | 0.478 | 0.040 | 0.021 | 0.143 | 0.122 | 0.056 | - | - | - | - |
| ZZ113 | 104 | 0.240 | 0.020 | 0.019 | 0.089 | 0.057 | 0.024 | 0.016 | 0.007 | 0.006 | 0.025 |
| ZZ109 | 13 | 0.131 | 0.015 | 0.085 | 0.086 | 0.125 | 0.035 | 0.022 | 0.025 | - | - |
|  | 34 | 0.071 | 0.009 | 0.016 | 0.053 | 0.105 | 0.032 | 0.011 | 0.009 | - | - |
| ZZ121 | 84 | 0.032 | 0.047 | 0.004 | 0.015 | 0.223 | 0.008 | 0.031 | 0.000 | 0.004 | 0.010 |
| ZZ132 | 7 | 0.086 | 0.008 | 0.023 | 0.098 | 0.095 | - | - | - | - | - |
|  | 13 | 0.172 | 0.010 | 0.038 | 0.492 | 0.286 | - | - | - | - | - |
|  | 27 | 0.134 | 0.013 | 0.014 | 0.207 | 0.178 | 0.042 | 0.020 | 0.006 | 0.019 | 0.011 |
| ZZ107 | 112 | 0.107 | 0.011 | 0.011 | 0.117 | 0.191 | 0.025 | 0.104 | 0.007 | 0.010 | 0.021 |
| ZZ120 | 114 | 0.078 | 0.005 | 0.006 | 0.044 | 0.032 | 0.012 | 0.036 | 0.004 | - | - |
| ZZ110 | 13 | 0.359 | 0.019 | 0.015 | 0.069 | 0.423 | 0.024 | 0.006 | 0.010 | - | - |
|  | 26 | 0.193 | 0.012 | 0.001 | 0.050 | 0.205 | 0.020 | - | - | - | - |
| ZZ105 | 39 | 0.019 | 0.013 | 0.006 | 0.030 | 0.064 | 0.042 | 0.057 | 0.009 | 0.028 | 0.055 |
| ZZ134 | 100 | 0.004 | 0.049 | 0.015 | 0.046 | 0.150 | 0.016 | - | - | - | - |
| ZZ119 | 112 | 0.217 | 0.018 | 0.041 | 0.117 | 0.247 | 0.059 | - | - | - | - |
| ZZ103 | 71 | 2.262 | 0.060 | 0.005 | 1.015 | 0.356 | 0.016 | 0.055 | 0.003 | - | - |
| ZZ116 | 102 | 0.084 | 0.019 | 0.009 | 0.164 | 0.068 | 0.016 | - | - | - | - |
| ZZ128 | 19 | 0.379 | 0.124 | 0.090 | 0.109 | 0.132 | 0.122 | 0.080 | 0.073 | - | - |
|  | 34 | 0.429 | 0.119 | 0.105 | 0.178 | 0.245 | 0.173 | 0.075 | 0.048 | - | - |
| **Cut off Value*** | **-** | **0.022** | **0.007** | **0.017** | **0.021** | **0.039** | **0.019** | **0.037** | **0.011** | **0.005** | **0.005** |
| ***Cut-off for a positive response was defined as the geometric mean T cell response plus 3 SE of 5 healthy subjects and 7 subjects enrolled but**  **confirmed ZIKV-negative.**  **Cells with dashes in the table indicates that the peptide pool was not tested for that participant (due to limitations in PBMC availability).**  **DPO, days post onset of symptoms.** | | | | | | | | | | | |
